# Effect of traditional roughage-based or limit-fed, high-energy diets on growth performance and digestion in newly received growing cattle and subsequent implications on feedlot growth performance and carcass characteristics

**DOI:** 10.1093/tas/txae082

**Published:** 2024-05-08

**Authors:** Morgan A Scilacci, Evan C Titgemeyer, Zachary M Duncan, Tyler J Spore, Sean P Montgomery, Travis G O’Quinn, Anthony J Tarpoff, William R Hollenbeck, Dale A Blasi

**Affiliations:** Department of Animal Sciences and Industry, Kansas State University, Manhattan, KS 66506, USA; Department of Animal Sciences and Industry, Kansas State University, Manhattan, KS 66506, USA; Department of Animal Sciences and Industry, Kansas State University, Manhattan, KS 66506, USA; Innovative Livestock Services, Inc., Great Bend, KS 67530, USA; Corn Belt Livestock Services, Papillion, NE 68046, USA; Department of Animal Sciences and Industry, Kansas State University, Manhattan, KS 66506, USA; Department of Animal Sciences and Industry, Kansas State University, Manhattan, KS 66506, USA; Department of Animal Sciences and Industry, Kansas State University, Manhattan, KS 66506, USA; Department of Animal Sciences and Industry, Kansas State University, Manhattan, KS 66506, USA

**Keywords:** ad libitum, coproduct, limit-feeding, liver abscesses, ruminal health

## Abstract

The objective was to determine the effects of ad libitum-fed roughage-based diets or limit-fed high-energy diets on growth performance, behavior, health, and digestion in newly received growing cattle and subsequent implications on feedlot growth performance and carcass characteristics. In experiment 1, 409 crossbred heifers (initial body weight [BW] = 279 ± 24 kg) in 32 pens were used in a randomized block design. Heifers were fed one of two dietary treatments: a total mixed ration with 0.99 Mcal net energy for gain (NE_g_)/kg dry matter (DM) fed ad libitum (0.99AL) or 1.32 Mcal NE_g_/kg DM limit-fed at 85% of intake of heifers fed 0.99AL (1.32LF85%). Both diets contained 40% DM as a branded wet corn gluten feed. In experiment 2, 370 crossbred heifers (initial BW = 225 ± 20 kg) were used in a randomized block design and were fed a diet formulated to contain 0.99 Mcal of NE_g_/kg DM for ad libitum intake or a diet formulated to contain 1.32 Mcal of NE_g_/kg DM and fed at 2.2% of BW daily (DM basis; 1.32LF2.2). For experiments 1 and 2, treatment integrity was maintained through the finishing phase where cattle were fed a common diet. Cattle were sorted by BW into heavy and light groups prior to finishing, with light cattle fed longer than heavy cattle to reach similar harvest BW. In experiment 3, eight ruminally cannulated heifers (average BW = 305 ± 23 kg) were used in a 2-period cross-over design and fed treatments from experiment 1 to assess digestibility and ruminal fermentation characteristics. Gain:feed was 47% and 35% greater (*P* < 0.01) in experiments 1 and 2, respectively, for limit-fed heifers compared with 0.99AL heifers. Rumination time was greater (*P* < 0.01) for 0.99AL compared with limit-fed treatments in experiments 1 and 2. Activity was greater (*P* < 0.01) for 1.32LF2.2 than for 0.99AL in experiment 2. In experiment 1, more (*P* = 0.03) carcasses from light-sort heifers than carcasses from heavy-sort heifers had livers with large, active abscesses. In experiment 2, finishing phase morbidity was greater (*P* < 0.01) for 1.32LF2.2 than for 0.99AL. Light-sort groups had fewer (*P* < 0.01) edible livers than heavy-sort groups, suggesting that greater number of days on feed may increase the risk of liver abscess prevalence and condemnation. In experiment 3, apparent total-tract DM and organic matter digestibilities were greater (*P* < 0.01) for 1.32LF85% than for 0.99AL. Overall, dietary treatments during the growing phase had little carryover effect on feedlot growth performance, carcass characteristics, or liver abscesses prevalence at harvest.

## Teaser

We fed growing heifers a traditional high-roughage diet for ad libitum intake or a high-energy limit-fed diet and measured performance during the growing and finishing periods as well as carcass characteristics following harvest. Limit-feeding a high-energy diet during the growing period improved feed efficiency and had minimal effects of finishing growth performance, carcass characteristics, or liver abscess prevalence compared with feeding a high-roughage diet for ad libitum intake during the growing period.

## Introduction

Transport of young growing cattle to grower or feed yards leads to periods of feed and water deprivation, reducing subsequent feed intake and performance for several days following realimentation (Hutcheson and Cole, 1986). The use of roughages in traditional growing programs promotes dry matter (DM) intake and minimizes metabolic upsets (Lofgreen et al., 1975). However, greater intake of roughages comes at the expense of decreased feed conversion and elevated feed costs, resulting in unrecoverable economic losses (Richeson et al., 2019). By the same token, increasing energy density or modifying energy sources in the diet, namely via nonstructural carbohydrates, increases the risk for digestive disorders including ruminal acidosis and liver abscesses (Owens et al., 1998).

Limit-feeding, a well-documented nutritional strategy for growing cattle, offers attractive opportunities in some situations for enhanced growth performance by improving feed efficiency and health detection, decreasing cost of gain, and easing transitions to finishing diets (Galyean et al., 1999). Loerch (1990) reported that the growing strategy (high-roughage ad libitum vs. high-concentrate limit-fed) did not influence finishing phase performance or carcass characteristics. Drouillard et al. (1991) reported that limit-fed animals experience compensatory gains following a period of energy restriction. As a result, limit-fed growing strategies used prior to the finishing phase may alter the deposition of intramuscular fat, a significant factor determining final carcass value and quality (Lancaster et al., 2012). Corn co-products have been utilized in limit-fed growing diets (Montgomery et al., 2003; Felix et al., 2011). In recent studies, limit-fed diets that contained highly fermentable fiber in comparison to roughage-based diets had no deleterious effects on health during the growing phase (Spore et al., 2018, 2019).

Three experiments were conducted to compare two growing diet strategies; a high-roughage diet fed for ad libitum intake vs. a high-energy limit-fed diet. The objectives of experiments 1 and 2 were to evaluate the effects of growing strategy on growth performance and behavior during the growing period and to determine if growing strategy influenced subsequent feedlot performance and carcass characteristics. The objective of experiment 3 was to measure apparent diet digestibility and ruminal fermentation characteristics of heifers fed a high-roughage diet for ad libitum intake or limit-fed a high-energy diet.

## Materials and Methods

The Kansas State University Institutional Animal Care and Use Committee approved all animal handling and animal care practices used in our experiments. All animal procedures were conducted in accordance with the Guide for the Care and Use of Animals in Agricultural Research and Teaching (FASS, 2020).

### Experiment 1

#### Growing phase cattle management

Four-hundred nine weaned, crossbred heifers (body weight [BW] = 279 ± 24 kg) were purchased at auction markets in Texas and New Mexico, assembled at 2 different farms approximately 145 km southwest of Amarillo, TX, then shipped 917 km to the Kansas State University Beef Stocker Unit on May 28, 2019. On arrival, cattle were individually weighed, visually assessed for physical injuries or disease, and assigned a visual number ear tag. Prior to assignment to the experimental pen, cattle had access to long-stem hay and water via automatic waterers (Lil’ Spring 3000; Miraco Livestock Water Systems, Grinnell, IA). The following day (day 0) all cattle were individually weighed and given a pen assignment ear tag and an electronic identification ear tag. Additionally, all cattle received a modified-live vaccine (Bovishield Gold 4; Zoetis, Parsippany, NJ) to protect against infectious bovine rhinotracheitis, bovine viral diarrhea types 1 and 2, and parainfluenza type 3. Due to extensive vaccination history, no other vaccines were administered. Heifers were allocated by increasing BW on days −1 to 16 treatment blocks, each containing two pens, with approximately equal numbers of heifers in each block; then, a serpentine method was used within each block to allocate heifers by BW to pens. Each block contained 2 treatment pens with 13 or 14 heifers per pen. Heifers were used in a randomized complete block design, and pen was the experimental unit. Pens (9.1 × 15.2 m) were soil-surfaced with concrete bunks of 9.1 m in length attached to a 3.6-m concrete apron.

Two experimental dietary treatments were formulated (Table 1): 0.99 Mcal of net energy for gain (NE_g_)/kg DM fed for ad libitum intakes (0.99AL) or 1.32 Mcal NE_g_/kg DM limit-fed at 85% of 0.99AL intakes (1.32LF85%). Both diets contained 40% wet corn gluten feed (Sweet Bran; Cargill Animal Nutrition, Blair, NE) on a DM basis. Cattle were fed once daily at 0700 hours using a feed wagon with 2.3 kg breaks (Roto-Mix model 414-14B, Dodge City, KS). Bunks were visually assessed each morning at 0600 hours, and estimated orts were recorded. Daily orts for 0.99AL pens were visually targeted to 9 kg. On a weekly basis, orts were collected from each bunk, weighed on a small portable scale (model iGB; Ishida, Kyoto, Japan) to assess 0.99AL consumption, and returned to the bunk. Feed offered to 1.32LF85% pens were targeted at 85% of consumption by the ad libitum-fed pen in the same block. Limit-fed pens always consumed their daily allotment, requiring 3 to 4 h to reach an empty bunk. Feed samples were collected weekly and frozen at −20 °C. At the conclusion of the study, feed samples were thawed, mixed, subsampled, and frozen for nutrient analyses.

**Table 1.** Ingredient and analyzed nutrient composition of experimental diets fed during growing phase (experiments 1 to 3)

	Diet		
Item	0.99AL^a^	1.32LF^b^	GIT equilibration^c^
Ingredient, % of dry matter			
Dry-rolled corn	8.6	38.8	23.8
Wet corn gluten feed^d^	40.0	40.0	40.7
Long-stem alfalfa	22.5	6.5	14.2
Chopped prairie hay	22.5	6.5	14.4
Supplement^e^	6.4	8.2	6.9
Nutrient, % of dry matter			
Experiment 1			
Dry matter, % as fed	74.5	73.3	—
Organic matter	91.5	94.1	—
Crude protein	15.9	15.1	—
Starch	13.0	33.5	—
Neutral detergent fiber	36.4	24.3	—
Acid detergent fiber	18.1	9.8	—
Calcium	1.1	0.8	—
Phosphorus	0.6	0.6	—
Experiment 2			
Dry matter, % as fed	74.7	74.2	74.5
Organic matter	85.3	93.7	92.9
Crude protein	15.8	15.1	16.3
Starch	10.0	29.3	19.1
Neutral detergent fiber	40.8	25.7	33.6
Acid detergent fiber	20.8	9.9	15.9
Calcium	1.2	1.1	1.0
Phosphorus	0.5	0.6	0.6
Experiment 3			
Dry matter, % as fed	73.1	72.7	—
Organic matter	91.3	94.5	—
Crude protein	17.0	15.5	—
Starch	11.9	33.0	—
Neutral detergent fiber	36.4	24.6	—
Acid detergent fiber	18.3	9.9	—
Calcium	0.8	0.6	—
Phosphorus	0.6	0.7	—

^a^Formulated using ingredient compositions from NASEM (2016) to 0.99 Mcal NE_g_/kg DM and fed for ad libitum intake.

^b^Formulated using ingredient compositions from NASEM (2016) to 1.32 Mcal NE_g_/kg DM and limit-fed at 85% of 0.99AL treatment DM intake in experiment 1, at 2.2% of BW daily on a DM basis in experiment 2, and at 85% of pretrial 0.99AL DM intake in experiment 3.

^c^Gastrointestinal tract-fill equilibration diet was limit-fed at 2.5% of BW daily on a DM basis for the final 14 d of the backgrounding phase in experiment 2.

^d^Sweet Bran, Cargill Animal Nutrition, Blair, NE.

^e^Supplement pellet was formulated to contain (DM basis) 9.2% crude protein, 1.5% crude fat, 7.4% calcium, 0.22% phosphorus, 4.62% salt, 0.50% potassium, 331 mg/kg monensin, and 60.1 mg/kg diflubenzuron.

A scale (Rice Lake Weighing Systems, Rice Lake, WI) was used to measure pen BW on a weekly basis from days 14 to 84. Individual heifer BW was also measured on days 0 and 84. Final performance data was calculated using pen BW over 2-wk intervals from days 0 to 84. To measure shrunk final BW, pens in 8 blocks were withheld access to feed on day 83. Shrunk pen BW was measured on the following day (day 84) prior to feeding. On day 84, pens in the remaining 8 blocks were withheld access to feed, and shrunk pen BW was measured prior to feeding on day 85.

All animals were observed twice daily for signs of lameness or morbidity, including depression, nasal or ocular discharge, and anorexia. Any animals displaying these symptoms were promptly removed from their pen for further observation and, if necessary, treatment. Rectal temperature was measured, and a clinical illness score was determined chute-side: 1, normal, healthy animal; 2, slightly ill with mild depression or gauntness; 3, moderately ill with severe depression, labored breathing, and nasal or ocular discharge; 4, severely ill, near death, and showing minimal response to human approach. Heifers pulled from their pen exhibiting a rectal temperature above 40°C and clinical illness score ≥ 2 were treated with a florfenicol and flunixin meglumine combination (Resflor Gold; Merck Animal Health, Madison, NJ), enrofloxacin (Baytril 100; Bayer Livestock, Shawnee, KS), or oxytetracycline (Biomycin 200; Boehringer Ingelheim Animal Health, Duluth, GA) and returned to their home pen. Upon receiving a third treatment, heifers were considered chronic and removed from the study.

On day 84, trained technicians from the Cattle Performance Enhancement Company (Oakley, KS) performed an ultrasound evaluation of each heifer at the top backfat line and over the ribs to estimate backfat thickness and intramuscular fat deposition. Muscle depth was measured from the bottom backfat line to the top of the rib bones.

#### Rumination and activity

All heifers were outfitted with a 3-axial sensory accelerometer ear tag (Allflex Livestock Intelligence, Madison, WI; Wolfger et al., 2015). Tags continuously recorded rumination and activity in 2-h time increments throughout the growing phase of the study. Rumination measurements include time spent masticating or ruminating. Activity measurements included all other movements besides mastication and rumination. Rumination and activity measurements prior to day 10 were not analyzed to allow time for cattle to adapt to the facilities. Due to antenna reception issues, all data from day 23 and data from days 24 to 34 for three pens were removed from the analysis; complete data were collected after day 34.

#### Finishing phase and carcass evaluation

To prepare for and facilitate shipping to the feed yard (Pratt Feeders, Pratt, KS) for the finishing phase, all cattle received 1 of 4 color-coded tags based on growing phase dietary treatment (0.99AL or 1.32LF85%) and sort group, which was determined by day 84 pen BW (light-sort or heavy-sort). Sixteen pens (8 per treatment) were designated light-sort (BW = 354 ± 19 kg), and 16 pens (8 per treatment) were designated heavy-sort (BW = 393 ± 17 kg). Because cattle were initially allocated to growing phase treatment blocks based on BW, the allocation of pens of heifers to different sort groups was largely, but not exclusively, dependent on the blocking based on the initial BW of the cattle. Cattle continued to be fed their respective treatment diets until shipping.

On days 90 and 91, all cattle were shipped in their respective treatment sort groups 303 km to a feed yard (Pratt Feeders, Pratt, KS) and grouped in four pens (approximately 100 cattle/pen) according to growing phase dietary treatment and sort group. All animals were processed and fed according to standard feedlot protocols for the duration of the finishing period. Cattle were started on a diet containing 28.6% hay and stepped up using 6 transition diets to a final finishing diet over 20 d (Table 2). Cattle were fed three times daily and feed calls were targeted so that only crumbs remained the following day. At day 61 of finishing, steam-flaked wheat in the diet was replaced with steam-flaked corn (Table 2). Feed samples were collected approximately once per month during the finishing phase and frozen at −20 °C for nutrient analysis.

**Table 2.** Ingredient and analyzed nutrient composition of finishing phase diets (experiment 1)

	Days of finishing phase			
Item	Initial^a^	21-60	61-132	133-end
Ingredient, % of dry matter				
Hay^b^	28.6	9.1	8.9	9.9
Steam-flaked corn	40.7	52.6	76.0	75.0
Steam-flaked wheat	21.9	27.2	—	—
Dried distiller’s grain	3.4	3.4	3.4	3.4
Fat	1.2	2.5	2.5	2.5
Corn syrup	—	—	3.8	3.8
Liquid supplement	4.3	5.2	5.4	5.4
Nutrient, % of dry matter				
Dry matter, % as fed	79.9	79.4	78.4	77.4
Crude protein	14.0	12.8	13.0	12.3
Fat	4.0	5.4	5.7	5.7
Neutral detergent fiber	20.1	12.5	13.0	12.4
Calcium	0.7	0.6	0.6	0.6
Phosphorus	0.2	0.3	0.3	0.3

^a^Initial diet at feedyard. Diet was transitioned over 20 d to the finishing diet fed from days 21 to 60.

^b^Blended ingredient: 80% chopped alfalfa and 20% cane (sorghum–sudangrass hybrid) hay.

Cattle were marketed by sort-group based on the feedyard’s assessment of optimal economic benefit and transported 122 km to a commercial abattoir (National Beef, Dodge City, KS) on January 14, 2020 (heavy-sort) and February 4, 2020 (light-sort). Finishing growth performance data was calculated by using individual shrunk BW collected on days 84 and 85 of the growing phase as the initial BW. Data from cattle that died during the finishing phase were excluded from finishing phase analyses. Final BW was calculated by dividing hot carcass weight by average dressing percentage. Carcass characteristics and liver scores (Brown and Lawrence, 2010) were obtained by trained personnel from the Beef Carcass Research Center at West Texas A&M University in Canyon, TX.

#### Nutrient analysis

All growing phase feed samples were delivered frozen to a commercial laboratory (SDK Laboratories, Hutchinson, KS) for nutrient analysis. Nutrient analyses of finishing diets (Table 2) were conducted by a commercial laboratory (Midwest PMS, Firestone, CO).

### Experiment 2

#### Growing phase cattle management

Crossbred heifers (*n* = 370; initial BW = 225 ± 20 kg) were purchased and assembled at an auction facility in Dickson, TN, and shipped 1,067 km to the Kansas State University Beef Stocker Unit on 4 trucks, each providing cattle for one block, on March 11, 2020, March 12, 2020, March 17, 2020, and March 19, 2020. Experimental design was a randomized complete block, and experimental unit was pen. On arrival (day −1) cattle were individually weighed and assigned a visual number ear tag. All cattle were ear-notched to provide samples to test for individuals persistently infected with bovine viral diarrhea virus. For 3 loads, notch samples were placed on ice and taken to the Kansas State Veterinary Diagnostics Laboratory (Manhattan, KS) for analysis, and for one load notch samples were tested using a test kit (SNAP BVDV Antigen test; IDEXX Laboratories, Westbrook, ME); one animal tested positive and was excluded from the experiment. Cattle had ad libitum access to long-stem prairie hay and water via automatic waterers (Lil’ Spring 3000; Miraco Livestock Water Systems, Grinnell, IA) prior to allocation to experimental pens on day 0.

Twenty-four hours after arrival (day 0), cattle were individually weighed and received visual and electronic identification ear tags. Cattle received a 7-way clostridial vaccine (Caliber 7; Boehringer Ingelheim Animal Health, Duluth, GA) and Titanium 5 (Elanco Animal Health, Greenfield, IN), a modified-live vaccine for protecting against infectious bovine rhinotracheitis, bovine viral diarrhea types 1 and 2, and parainfluenza type 3. Additionally, cattle received Nuplura PH (Elanco Animal Health, Greenfield, IN) for protection against *Mannheimia haemolytica*, and tulathromycin (Draxxin; Zoetis, Parsippany, NJ), a macrolide antibiotic. Upon completion of processing, heifers within truckload were distributed to four pens using a serpentine method based on day −1 BW, and two pens from each block were randomly assigned to each of two dietary treatments. Heifers were housed in 16 soil-surfaced pens (18.2 m × 15.2 m) with four pens per block. About 20 to 25 heifers were allocated to each treatment pen, and this was dependent on how many cattle were in each truckload. Cattle were revaccinated on day 14 using Titanium 5.

The two experimental treatment diets (Table 1), both containing 40% wet corn gluten feed (Sweet Bran; Cargill Animal Nutrition) on a DM basis, were identical to experiment 1, except the limit-fed treatment (1.32LF2.2) was fed at 2.2% of BW daily on a DM basis. Heifers were fed once daily at 0700 hours using the same mixing wagon as experiment 1. Bunks were visually observed, and orts were estimated at 0630 hours. Orts for the 0.99AL treatment were targeted at 9 kg for each pen. Treatment diets were fed for 84 d for 2 blocks and for 91 d for 2 blocks. A common gastrointestinal tract-fill equilibration diet formulated to contain 1.17 Mcal NE_g_/kg DM (Table 1) was fed at 2.5% of BW daily on a DM basis for 14 d beginning on day 84 or day 91 of the backgrounding phase. A scale (Rice Lake Weighing Systems) was used to measure weekly pen BW, which was utilized to adjust feed offerings and calculate pen performance. Individual BW was measured on arrival, at revaccination, and at the end of the gut-fill equilibration period. Feed samples were collected weekly and frozen at −20 °C. At the conclusion of the study, feed samples were thawed, mixed, subsampled, and frozen until delivered frozen to a commercial laboratory for nutrient analysis. All animals were observed twice daily for signs of lameness or morbidity, including depression, nasal or ocular discharge, and anorexia and treated using the protocol described in experiment 1.

#### Rumination and activity

Each heifer was outfitted with a 3-axial accelerometer ear tag (Allflex Livestock Intelligence) on day 0 of the experiment. Due to data collection glitches, only data after day 50 was included in the analysis. Tags were removed prior to the 14-d gastrointestinal tract-fill equilibration period at the conclusion of the trial.

#### Finishing phase and carcass evaluation

Prior to shipment for finishing, cattle were sorted into a heavy-sort or light-sort based on final individual BW measured on day 98 or day 105, depending on the block. Sort group BW thresholds were established for each experimental treatment group (0.99AL: BW = 362.8 kg; 1.32LF2.2: BW = 358.3 kg) to obtain an approximately equal number of cattle in each of four groups. Cattle were loaded into trucks to maintain backgrounding treatment and sort group integrity and shipped 303 km to a commercial feed yard (Pratt Feeders, Pratt, KS). Upon arrival at the feedlot, cattle were placed in four pens (approximately 100 heifers/pen) according to backgrounding treatment and sort group. All cattle followed the same feedlot protocols as experiment 1 and were fed diets similar to those described for experiment 1 (Table 2), but feed samples were not collected for nutrient analysis. Cattle were marketed by sort group and transported 122 km to a commercial abattoir (National Beef, Dodge City, KS) on November 17, 2020 (heavy-sort) and January 12, 2021 (light-sort), and carcass characteristic data were collected. Finishing growth performance was calculated by using individual shrunk BW collected after the gastrointestinal tract-fill equilibration period at the end of the growing phase as initial BW. Final BW was calculated by dividing hot carcass weight by average dressing percentage collected at the abattoir. Cattle that died during the finishing phase were excluded from the analyses. Three heifers were removed from carcass data analyses due to inability to identify original treatment pen during the backgrounding phase; one heifer was removed due to incorrect feedlot pen placement.

##### Net Energy Calculations

In experiments 1 and 2, performance data were used to calculate net energy for maintenance (NEm) and NE_g_ as described by Galyean (2019) using equations from NRC (1996). Initial BW in both experiments were pencil shrunk by 4% to account for gastrointestinal tract fill when cattle were offered ad libitum access to hay; final BW were not shrunk.

### Experiment 3—Intake and Digestibility Study

#### Cattle Management

Eight ruminally cannulated crossbred Angus heifers (average BW = 305 ± 23 kg) were used in a cross-over design with two consecutive 15-d periods. The experimental unit was animal within the period. Due to cannula issues, data for one heifer were removed on day 15 of the first period. Experimental diets were the same as experiment 1 (Table 1). When feed was mixed for experiment 1, feed was removed from the beginning of the wagonload for experiment 3, and samples separate from those from experiment 1 were obtained for nutrient analysis.

Heifers were housed individually in soil-surfaced, outdoor pens (6.1 m × 12.2 m). Each pen had access to a manually filled water tank. Cattle were fed once daily at 1000 hours. Each 15-d period included 10 d for diet adaptation, 4 d for fecal sampling, and 1 d for ruminal sampling. All cattle were offered the 0.99AL treatment diet for 7 d prior to study initiation to acclimate and determine ad libitum intakes. Orts for heifers receiving 0.99AL were targeted at 1.8 kg/d during diet adaption and sampling. Cattle receiving the limit-fed diet (1.32LF85%) were restricted to 85% of their own reference 0.99AL DM intake determined prior to study initiation. Prior to the start of the experiment, an indwelling rumen pH bolus (smaXtec, Graz, Austria) was inserted through the ruminal cannula. Each bolus measured ruminal pH every 10-min. Only data collected from days 11 to 15 of each period were used in the analysis. Prior to analysis, ruminal pH measurements for each heifer within period were averaged across hour.

On days 4 to 14, 10 g of chromic oxide (Cr_2_O_3_) was top-dressed and hand-mixed into each total mixed ration to serve as an indigestible marker for determining apparent total-tract diet digestibility. Feed samples were collected on days 10 to 14. Orts were collected on days 11 to 14 for each animal. Fecal samples were collected from the rectum of each animal on days 11 to 14 at 8-h intervals after feeding with sampling time advanced by 2 h each day, so each 2-h interval of the day was represented. Immediately following collection, all samples were frozen at −20 °C. Following study completion all feed, fecal, and ort samples were thawed, mixed, subsampled, and refrozen by animal within period.

On day 15 of each period, four locations in the rumen were sampled prior to feeding, and at 2, 4, 6, 8, 12, 18, and 24 h after feeding to determine ruminal volatile fatty acid (VFA) profile and ammonia concentration. Approximately 100 mL of ruminal fluid was immediately strained through 8 layers of cheesecloth. Strained ruminal fluid (1 mL) was pipetted into four 2-mL microcentrifuge tubes each containing 0.25 mL of 25% (wt/vol) *m*-phosphoric acid, then frozen at −20 °C. Following collection of 0 h samples, 3 g of Co-EDTA dissolved into 200 mL of distilled water was dosed through the ruminal cannula. At 2, 4, 6, 8, 12, 18, and 24 h sampling times, 15 mL of ruminal fluid was pipetted into 20-mL scintillation vials for later measurement of Co concentrations for estimation of liquid passage rate and ruminal liquid volume. After collection all ruminal fluid samples were frozen at −20 °C.

#### Laboratory analysis and calculations

Composited samples of feed, orts, and feces were delivered frozen to a commercial laboratory (SDK Laboratories, Hutchinson, KS) for analysis of DM, organic matter, neutral detergent fiber, acid detergent fiber, crude protein, and starch. Dry sample aliquots of orts and feces were ground to pass through a 1-mm screen. To prepare ruminal fluid samples for Co analysis, 5 mL was centrifuged at 25,000 × *g* for 25 min at 4 °C, and supernatant was analyzed by atomic absorption spectrophotometry (Perkin Elmer AAnalyst 100; PerkinElmer, Waltham, MA). Liquid passage rate was estimated by regressing the natural logarithm of cobalt concentration for samples collected from 2 to 18 h after Co-EDTA dosing against time for each animal in each period using the NONLINEAR procedure in SAS. Liquid passage rate was determined as the negative slope from the regression, and ruminal liquid volume was calculated by dividing the amount of Co dosed by the ruminal concentration of Co at 0 h predicted from the y-intercept of the regression.

To prepare acidified ruminal fluid samples for analysis of VFA and ammonia concentrations, samples were centrifuged at 17,000 × *g* for 30 min at 4 °C. Supernatant was analyzed for VFA by gas chromatography as described by Vanzant and Cochran (1994). Analysis of ammonia concentration used procedures of Broderick and Kang (1980).

To calculate apparent total-tract diet digestibility, wet fecal samples, and dried, ground ort samples were analyzed for Cr by atomic absorption spectrophotometry (Perkin Elmer AAnalyst 100; PerkinElmer, Waltham, MA) following sample preparation as described by Williams et al. (1962). Fecal output (g/d) was estimated by dividing Cr intake (g/d) by Cr concentration in the feces (g Cr/g fecal DM).

### Statistical Analyses

For experiments 1 and 2, growing phase growth performance, net energy calculations, and behavior (rumination and activity) data were analyzed as randomized block designs using the MIXED procedure in SAS (v9.4, SAS Institute Inc., Cary, NC). Pen was the experimental unit. Dietary treatment and block were included as fixed effects in the model. Behavior data were analyzed with block, dietary treatment, day, and dietary treatment × day interaction included as fixed effects in the model, using the day as the repeated measure and pen as the subject. For rumination data, the covariance structures were autoregressive in experiment 1 and compound symmetry in experiment 2, based on the Akaike information criterion. For activity, the selected covariance structures were spatial power for experiment 1 and autoregressive for experiment 2. In addition, a behavior data set which was averaged over days 10 to 83 was analyzed with block, dietary treatment, hour, and treatment × hour interaction included as fixed effects in the model. The MIXED procedure in SAS was used to analyze finishing phase growth performance and carcass data from individual heifer data with the fixed effects of backgrounding diet, sort group, and backgrounding diet × sort group interaction. Block and backgrounding diet × pen (from the growing phase) interaction were included as random effects. For finishing phase morbidity, mortality, liver characteristics, and USDA quality grades percentages of heifers within each category were calculated for each pen and analyzed with a model including backgrounding diet, sort group, and backgrounding diet × sort group interaction as fixed effects and block as a random effect.

In experiment 3, all data were analyzed with the MIXED procedure of SAS as a cross-over design. Criteria without repeated measures were analyzed with treatment and period as fixed effects in the model, and animal as a random effect. For ruminal VFA and ammonia concentrations, repeated measures analyses were used, with fixed effects including treatment, period, hour, and treatment × hour interaction; animal was included as a random effect. The repeated measure was hour, with subject being animal × period, and the covariance structure was selected for each dependent variable based on the Akaike information criterion. For analysis of ruminal pH data collected by smaxTec boli, treatment, period, hour, and treatment × hour interaction were included as fixed effects in the model; animal and day were included as random effects. Hour served as the repeated measure, with subject being animal × period × day. The covariance structure selected was spatial power, based on the Akaike information criterion. Significance was declared at *P* ≤ 0.05, and tendencies at *P* ≤ 0.10.

## Results and Discussion

### Experiment 1

#### Growing phase growth performance and health

Results of growing performance, ultrasound, and behavior parameters are presented in Table 3. Overall, gain:feed was 47% greater (*P* < 0.01) for heifers fed 1.32LF85% compared with heifers fed 0.99AL. The better efficiency for 1.32LF85% than for 0.99AL was the result of 15% greater (*P* < 0.01) ADG and 22% lower (*P* < 0.01) DM intake. Cattle fed 0.99AL had less (*P* < 0.01) muscle depth and less (*P* < 0.01) backfat compared with cattle fed 1.32LF85%. Differences in muscle depth and back fat at the conclusion of the growing period were expected considering the differences in ADG between treatments. Heifers exhibited few clinical signs of morbidity, and mortality was low (Table 3).

**Table 3.** Effect of ad libitum high-roughage or limit-fed high-energy diets in the backgrounding phase on performance and behavior (experiment 1)

	Diet^a^			
Item	0.99AL	1.32LF85%	SEM^b^	*P* value
Number of pens	16	16		
Number of animals	205	204		
Body weight, kg				
Day 0	280.1	278.9	0.93	0.36
Day 14	293.9	288.0	1.17	<0.01
Day 28	316.6	319.5	1.59	0.23
Day 42	341.3	339.1	1.28	0.23
Day 56	350.9	355.7	1.89	0.09
Day 70	373.4	379.6	1.45	<0.01
Day 84	367.6	379.6	1.66	<0.01
Average daily gain, kg/d				
Days 0 to 14	0.99	0.65	0.04	<0.01
Days 0 to 28	1.30	1.45	0.04	0.02
Days 0 to 42	1.46	1.43	0.02	0.50
Days 0 to 56	1.26	1.37	0.03	0.02
Days 0 to 70	1.33	1.44	0.02	<0.01
Days 0 to 84	1.04	1.20	0.02	<0.01
Dry matter intake, kg/d				
Days 0 to 14	7.12	5.89	0.02	<0.01
Days 0 to 28	8.61	7.13	0.03	<0.01
Days 0 to 42	9.27	7.58	0.03	<0.01
Days 0 to 56	9.58	7.71	0.06	<0.01
Days 0 to 70	10.11	8.04	0.06	<0.01
Days 0 to 84	10.26	8.02	0.05	<0.01
Daily intake, % of body weight daily				
Days 0 to 14	2.48	2.08	0.01	<0.01
Days 0 to 28	2.89	2.38	0.01	<0.01
Days 0 to 42	2.99	2.45	0.01	<0.01
Days 0 to 56	3.04	2.43	0.02	<0.01
Days 0 to 70	3.10	2.44	0.02	<0.01
Days 0 to 84	3.18	2.44	0.02	<0.01
Gain:feed, kg/kg				
Days 0 to 14	0.140	0.111	0.01	<0.01
Days 0 to 28	0.152	0.204	0.01	<0.01
Days 0 to 42	0.158	0.194	0.01	<0.01
Days 0 to 56	0.133	0.179	0.01	<0.01
Days 0 to 70	0.132	0.180	0.01	<0.01
Days 0 to 84	0.102	0.150	0.01	<0.01
NE_m_, Mcal/kg dry matter^c^	1.24	1.62	0.01	<0.01
NE_g_, Mcal/kg dry matter^c^	0.68	1.01	0.01	<0.01
Morbidity, %				
Treated once or more	0.5	1.0	—	—
Treated twice	0.0	0.5	—	—
Mortality, %	0.5	1.9	—	—
Ultrasound measurements^d^				
Marbling score	4.78	4.92	0.04	0.02
Muscle depth, cm	5.36	5.72	0.05	<0.01
Backfat, cm	0.51	0.56	0.03	<0.01
Rumination, min/d^e^	444.3	407.2	3.27	<0.01
Activity, min/d^e^	319.8	317.8	1.40	0.33

^a^0.99AL = 0.99 Mcal NE_g_/kg DM fed for ad libitum intake. 1.32LF = 1.32 Mcal NE_g_/kg DM limit-fed at 85% of 0.99AL treatment DM intake.

^b^Largest SEM is reported.

^c^Net energy for maintenance (NE_m_) or net energy for gain (NE_g_) calculated from days 0 to 84 based on NRC (1996) equations.

^d^Measured via ultrasound on day 84. Marbling scores: 4.00 to 4.99 = select. 5.00 to 5.99 = low choice. Muscle depth is from the bottom backfat line to the rib bones. Backfat was measured at top of backfat line.

^e^Measured using 3-axial accelerometer ear tags (Allflex Livestock Intelligence, Madison, WI).

Improvements in growth performance for 1.32LF85% compared with 0.99AL agrees with a previous study with growing heifers that evaluated four dietary energy concentrations with correspondingly restricted intakes designed to program rate of gain at 1 kg/d (Spore et al., 2019). In that study, gain:feed was 22% greater for heifers fed a diet that contained 1.32 Mcal NE_g_/kg DM compared with those fed a diet that contained 0.99 Mcal NE_g_/kg DM, although ADG was not different between those treatments, reflecting that intakes were programmed to yield similar ADG. In contrast to Spore et al. (2019), ADG in our trial was greater (*P* < 0.01) for 1.32LF85% compared with 0.99AL. Greater ADG for heifers fed 1.32LF85% was likely due to greater net energy intake; by design, cattle fed 1.32LF85% had 13% greater intake of dietary NE_g_ than 0.99AL contemporaries (1.32 × 0.85/0.99 = 1.13). Net energy densities calculated from animal performance were lower than what was formulated in the diets, and the ratio of observed NE_g_ to formulated NE_g_ was slightly greater for 1.32LF85% than for 0.99AL. The 0.99AL diet yielded 0.68 Mcal NE_g_/kg DM based on cattle performance, and the 1.32LF85% diet yielded 1.01 Mcal NE_g_/kg DM. Possible reasons for lower NE values based on performance compared to diet formulation include pen conditions and heat stress, due to the experiment being conducted during a hot, wet summer. Thus, some energy may have been shifted away from growth in support of greater maintenance activities.

We attempted to equalize gut fill between treatments by fasting animals for a day prior to weighing. It is notable that the 0.99AL cattle demonstrated a loss of BW between days 70 and 84, whereas the 1.32LF85% cattle maintained BW over this period; the resulting difference in BW gain over the final 14 d would mostly be reflective of different changes in gut fill between the treatments because gains were roughly similar between the treatments between days 28 and 70.

 Hicks et al. (1990) observed up to a 10.9% improvement in feed effciency when cattle were limit-fed finishing diets, yet a direct comparison to our work is problematic, because our study used two distinct energy-intake regimens, thus widening differences in gain:feed. In another limit-feeding trial, Knoblich et al. (1997) examined 5 different growing–finishing systems of identical dietary composition using crossbred steers initially limit-fed high-energy diets to allow gains of 1.13 kg/d, then subsequently offered amounts to allow gains of 1.36 kg/d and, finally, fed ad libitum until slaughter. In accordance with our results, during the ad libitum feeding period, steers that had previously been limit-fed had greater ADG and gain:feed compared with steers fed ad libitum. However, gastrointestinal tract-fill may have inflated gains and efficiencies of previously limit-fed cattle in that experiment because limit-fed cattle would have had less gut fill prior to being fed ad libitum. Other studies confirm the interference of gut fill in calculating performance of limit-fed cattle (Coleman et al., 1995; Watson et al., 2013).

Berry et al. (2004) fed two different dietary energy/starch combinations to young growing cattle, and morbidity was not different between high- and low-energy treatments, but high-starch diets tended to increase morbidity. Although 1.32LF85% contained more starch than 0.99AL in our experiment, morbidity during the growing period was similar between 1.32LF85% and 0.99AL. Spore et al. (2018) reported no detrimental impacts of limit-feeding high-energy diets on antibody titer production even though ruminal pH briefly dropped into subacute acidotic ranges when newly received cattle were fed higher dietary energy concentrations at limited intakes.

#### Rumination and activity

**Figure 1.**
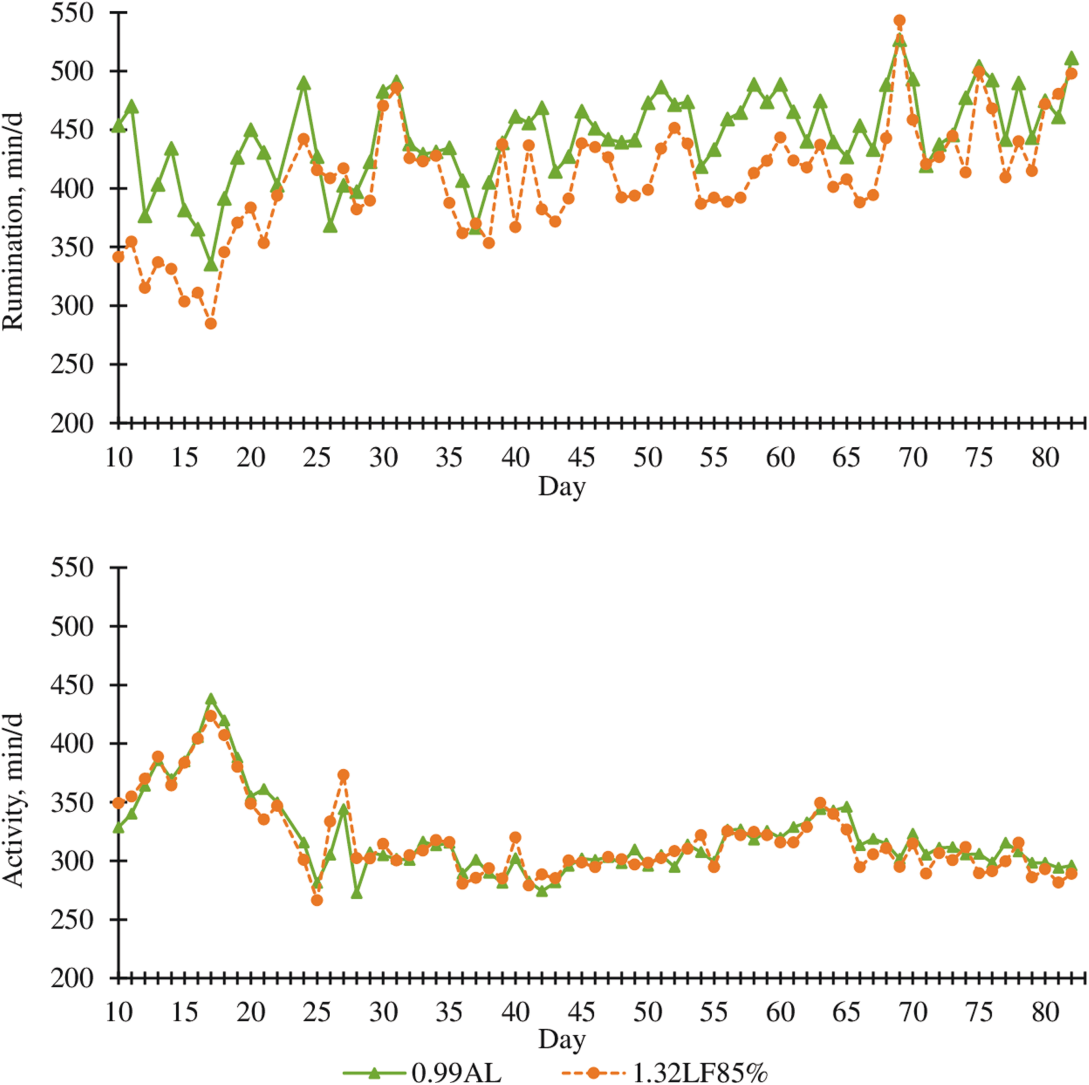
Effect of ad libitum high-roughage or limit-fed high-energy diets in the backgrounding phase on daily rumination and activity (experiment 1). 0.99AL (filled triangle) = 0.99 Mcal NE_g_/kg DM offered for ad libitum intake, *n* = 205; 1.32LF85% (filled circle) = 1.32 Mcal NE_g_/kg DM limit-fed at 85% of 0.99AL DM intake, *n* = 204. Rumination (top graph): diet, *P* < 0.0001; day, *P* < 0.0001; diet × day, *P *< 0.0001. SEM = 14.2. Activity (bottom graph): diet, *P* = 0.33; day, *P* < 0.0001; diet × day, *P* = 0.04. SEM = 8.1.

**Figure 2.**
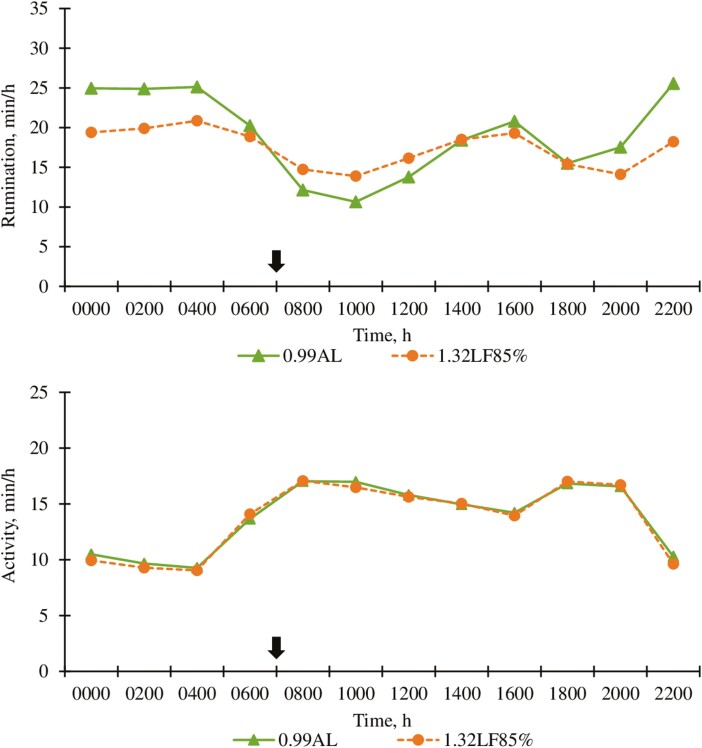
Effect of ad libitum high-roughage or limit-fed high-energy diets in the backgrounding phase on hourly rumination and activity (experiment 1). 0.99AL (filled triangle ) = 0.99 Mcal NE_g_/kg DM offered for ad libitum intake, *n* = 205; 1.32LF85% (filled circle) = 1.32 Mcal NE_g_/kg DM limit-fed at 85% of 0.99AL DM intake, *n* = 204. Arrows represent time of feeding (0700 hours). Rumination (top graph): diet, *P* < 0.0001; hour, *P* < 0.0001, diet × hour, *P* < 0.0001. SEM = 0.38. Activity (bottom graph): diet, *P* = 0.02; hour, *P* < 0.0001; diet × hour, *P* = 0.02. SEM = 0.17.

Cattle fed 1.32LF85% ruminated 37 min/d less (*P* < 0.01; Table 3) than cattle fed 0.99AL. A dietary treatment × day interaction (*P* < 0.01; Fig. 1) for rumination time was observed. Differences in rumination time between treatments were apparent between days 10 to 21 and days 40 to 68, but not over other portions of the trial; however, reasons for this interaction are not clear. A dietary treatment × hour interaction (*P* < 0.01; Fig. 2) was also observed for rumination time. Cattle fed 0.99AL spent more time ruminating during the overnight hours (*P* < 0.01; 2000 to 0600 hours), whereas 1.32LF85% of cattle ruminated more in the morning after feeding (*P* < 0.01; 0800 to 1200 hours). Jennings et al. (2020) reported that rumination time decreased as roughage inclusion in diet was reduced. Similarly, Gentry et al. (2016) and Weiss et al. (2017) observed a reduction in rumination time as corn stalk particle size was reduced. Greater roughage inclusion and larger particle sizes in 0.99AL compared with 1.32LF85% likely contributed to differences in rumination time between treatments.

Activity appeared greater during the first portion of the experiment and reached a nadir after 3 wk which was maintained over the remainder of the experiment (Fig. 1). Activity averaged over the trial did not differ (*P* = 0.33) between treatment groups, suggesting that energy savings as a result of reduced activity are not likely a major contributor to the improved efficiency for 1.32LF85% compared to 0.99AL. A treatment × day interaction (*P* = 0.04; Fig. 1) was observed for activity, but this appeared to be a result of random day-to-day variations between treatments. A dietary treatment × hour interaction (*P* < 0.02; Fig. 2) was detected for activity, but the differences over the day were relatively minor and unlikely to be of biological importance.

#### Finishing Phase Growth Performance and Carcass Characteristics.

Finishing growth performance is presented in Table 4. DM intake and gain:feed was assessed for numerical differences but not statistically analyzed because all cattle from each treatment were fed in a single pen. There were no significant interactions (*P* ≥ 0.17) between main effects of backgrounding diet and sort group for finishing growth performance. There was an effect of growing diet (*P* < 0.01) for initial BW and ADG, with 1.32LF85% cattle having greater initial BW than 0.99AL cattle, which was due to treatment differences that occurred in the growing phase. Cattle fed 0.99AL had 5.5% better (*P* = 0.03) ADG compared to 1.32LF85% cattle. No main effects (*P* ≥ 0.21) of growing diet were detected for days on feed, final BW, morbidity, or mortality. Main effects of sort group were detected for days on feed, with light-sort cattle requiring 21 more (*P* < 0.01) days on feed than heavy-sort cattle to reach a similar (*P* ≥ 0.24) target final BW. Heavy-sort cattle had greater (*P* < 0.01) initial BW by design, better (*P* = 0.03) ADG, and a tendency (*P* < 0.07) for less morbidity than light-sort cattle. No main effects of sort group (*P* ≥ 0.32) were detected for mortality, although the number of mortalities were low for all treatments. Heavy-sort cattle appeared to have greater DM intake than light-sort cattle, but gain:feed differences between growing diet/sort groups were minor.

**Table 4.** Effect of dietary treatment during the backgrounding phase and sort group during the finishing phase on finishing growth performance and carcass characteristics (experiment 1)

	Sort Group^1^							
	Heavy		Light					
	Backgrounding Diet^2^					*P* value^3^		
Item	0.99AL	1.32LF85%	0.99AL	1.32LF85%	SEM^4^	S	B	S × B
Number of pens	1	1	1	1				
Finishing phase performance								
Number of animals	102	100	102	101				
Days on feed	145	145	166	166	0.04	<0.01	0.99	0.67
Initial weight, kg	393.3	402.8	351.5	362.0	5.5	<0.01	<0.01	0.67
Final weight^5^, kg	590.0	585.6	571.4	581.0	8.8	0.24	0.70	0.31
Average daily gain, kg/d	1.42	1.31	1.32	1.29	0.03	0.03	0.03	0.17
Dry matter intake, kg/d	9.87	9.71	9.27	9.07	—	—	—	—
Gain:feed, kg/kg	0.144	0.135	0.142	0.142	—	—	—	—
Morbidity, %	6.9	2.1	14.1	9.5	3.7	0.07	0.21	0.98
Mortality, %	1.0	2.0	0.0	1.0	0.9	0.32	0.32	0.99
Carcass traits								
Number of animals	97	97	102	96				
Hot carcass weight, kg	384.3	384.6	369.3	378.0	5.7	0.09	0.31	0.34
Dressing percentage, %	65.14	65.68	64.62	65.06	—	—	—	—
Backfat, cm	1.69	1.91	1.61	1.68	0.05	<0.01	<0.01	0.11
USDA yield grade	2.45^a^	2.85^b^	2.52^a^	2.43^a^	0.08	0.03	0.09	<0.01
Marbling score^6^	520	546	546	527	10.2	0.73	0.76	0.02
Ribeye area, cm^2^	96.5^b^	94.8^b^	90.4^a^	97.0^b^	1.0	0.07	0.02	<0.01
USDA quality grade, %								
Select	3.1	2.0	7.8	9.4	1.7	<0.01	0.88	0.46
Choice	91.9	93.0	76.5	85.5	2.6	<0.01	0.07	0.16
Prime	5.9	4.9	14.0	5.2	2.3	0.13	0.02	0.06
Liver characteristics^7^, %								
Scars	36.1^ab^	48.5^b^	38.2^ab^	28.1^a^	5.2	0.15	0.82	0.04
Edible	87.7	92.7	78.4	71.9	3.5	<0.01	0.83	0.13
Telangiectasis	2.7	1.9	2.1	2.0	1.5	0.88	0.76	0.78
Liver score^8^, %								
0	88.8	92.7	83.2	81.4	3.6	0.03	0.77	0.44
A−	5.2	5.2	5.9	6.3	2.3	0.67	0.94	0.99
A	0.0	1.0	1.0	1.0	0.9	0.59	0.53	0.59
A+	6.2	1.0	9.8	11.5	3.3	0.03	0.52	0.25

^ab^Least square means in the same row with different superscripts are significantly different (*P* < 0.05).

^1^Sort groups for each backgrounding treatment were created prior to finishing phase. Heavy-sort groups were harvested after 145 d of finishing, and light-sort groups were harvested after 166 d of finishing.

^2^Diets offered during the backgrounding phase prior to the finishing phase. 0.99AL = 0.99 Mcal NE_g_/kg DM fed for ad libitum intake. 1.32LF = 1.32 Mcal NE_g_/kg DM limit-fed at 85% of 0.99AL treatment DM intake.

^3^S = sort group; B = backgrounding diet.

^4^Largest standard error of the means are reported.

^5^Final weight was calculated from hot carcass weight divided by average dressing percentage of each finishing pen.

^6^<400 = Select. 400 to 499 = low Choice. 500 to 599 = average Choice. 600 to 699 = high Choice.

^7^Liver scars indicate healed abscesses which were no longer active. Edible livers are acceptable for human consumption. Telangiectasis is a disease of the liver causing small blood-filled cavities with red or purple mottling to develop.

^8^0, no abscesses; A−, one or two small, active abscesses; A, two to four small, active abscesses each with a diameter less than 2.5 cm; A+, one or more large, active abscesses (Brown and Lawrence, 2010).

There were significant interactions detected between main effects of growing diet and sort group for carcass characteristics (Table 4). Carcasses from cattle fed 1.32LF85% in the growing phase and in the heavy-sort group had larger (*P* < 0.01) USDA yield grade scores than cattle in the other 3 groups. Carcasses from cattle fed 0.99AL in the growing phase and in the light-sort group had smaller (*P* < 0.01) ribeye areas than the other three groups. An interaction (*P* = 0.02) between growing diet and sort group was observed for marbling, because 1.32LF85% carcasses tended (*P* = 0.07) to have greater marbling scores than 0.99AL carcasses for the heavy-sort group but demonstrated lower marbling scores for the light-sort group. Carcasses from the 1.32LF85% treatment and in the light-sort group had less (*P* = 0.04) liver scarring than those from cattle fed 1.32LF85% and in the heavy-sort group, whereas cattle fed 0.99AL in the growing period had intermediate amounts of scarring regardless of which sort group they were in. No other significant interactions between main effects were detected in this experiment.

Carcasses of cattle that had been fed 1.32LF85% had greater (*P* < 0.01) backfat than those of cattle fed 0.99AL, but more (*P *= 0.02) 0.99AL carcasses graded USDA Prime than 1.32LF85% carcasses. There was a tendency (*P* = 0.07) for a greater percentage of 1.32LF85% carcasses to grade USDA Choice than 0.99AL carcasses.

Carcasses from heavy-sort heifers had greater (*P* < 0.01) backfat than carcasses from light-sort heifers. Fewer (*P* < 0.01) carcasses from heavy-sort heifers graded USDA Select than carcasses from light-sort heifers, whereas 11.5% more (*P* < 0.01) carcasses from heavy-sort heifers graded USDA Choice than carcasses from light-sort heifers. More (*P* < 0.01) carcasses from heavy-sort heifers had edible livers compared with carcasses from light-sort heifers. More (*P* = 0.03) carcasses from light-sort heifers than carcasses from heavy-sort heifers had livers with large, active (A+) abscesses. More (*P* = 0.03) carcasses from heavy-sort heifers had livers with no abscesses compared to carcasses from light-sort heifers. There was a tendency (*P* = 0.09) for carcasses from heavy-sort heifers to have greater hot carcass weights than carcasses from light-sort heifers.

Sainz et al. (1995) fed either forage diets containing alfalfa and oat straw for ad libitum intakes or concentrate diets containing rolled wheat and rolled corn for ad libitum or limited intakes during the growing phase (237 to 327 kg), followed by a finishing period (327 to 481 kg) when a concentrate-based diet was offered for ad libitum intake to all groups. Average daily gains during the finishing phase were greater for steers limit-fed a concentrate diet during the growing phase compared with steers fed a forage-based diet. Differences in BW gains during the finishing period were attributed to compensatory gains. In that study, fasted BW was measured and used to determine performance, but the authors did not specify how the fast was conducted. The authors did suggest that gastrointestinal tract fill after the growing phase was likely greater in forage-fed steers than in steers limit-fed concentrate.

Backfat at the completion of the finishing period was greater (*P* < 0.01) for 1.32LF85% compared with 0.99AL. In addition, a diet × sort group interaction (*P* < 0.01) was observed for ribeye area. Carcasses from heavy-sort heifers and carcasses from light-sort limit-fed heifers had greater ribeye areas compared with carcasses from light-sort heifers fed ad libitum. In contrast to our results, Murphy and Loerch (1994) reported reductions of carcass fat in all-concentrate fed steers with intakes restricted to 80% of ad libitum intakes throughout the growing and finishing period, but no differences in carcass characteristics due to intake restriction were observed in a subsequent experiment. Although 1.32LF85% intakes were restricted in our trial, energy supply was slightly greater for steers provided the limit-fed diet than for those with ad libitum access to the higher forage diet. Overall it appears that differences in the level of feed restriction may affect carcass characteristics.

Growing diet had no effect on liver characteristics. However, our results suggest that finishing phase sort group, confounded by days on feed and harvest date, affected final liver characteristics. In addition, heifer morbidity tended (*P *= 0.07) to be greater in the light-sort group compared with the heavy-sort group which may have also influenced liver abscess prevalence. Lighter cattle with access to high-energy diets fed for ad libitum intakes for longer finishing periods may be at greater risk for liver abscess development and liver condemnation. In our experiment, energy concentration and intake restriction were confounded. Schmidt et al. (2005) formulated diets to provide similar NE concentrations and metabolizable protein supplies at 90% and 80% of ad libitum intake by altering diet composition; restricted intake diets contained 3.9% and 6.3% more crude fat, respectively. Final BW was greater for 80% intake-restricted cattle than cattle with ad libitum intake, and they reported no effect of intake restriction for growing and finishing cattle on liver abscess prevalence.

Loerch (1990) reported that longer finishing periods of feeding high-concentrate diets increased incidence of liver abscesses. However, in contrast to our experiment, Loerch (1990) found cattle limit-fed a high-concentrate diet had greater liver condemnation compared with cattle fed a low-energy, silage-based diet for ad libitum intake. The limit-fed diets in their trial contained 80% high-moisture corn, so intake of fermentable starch was high. Limit-fed diets in our study contained modest concentrations of starch and high concentrations of fermentable fiber due to inclusion of wet corn gluten feed, which likely limited development of liver abscesses. Generally, growing phase concentrate concentration does not appear to affect liver condemnation, whereas finishing phase concentration does (Loerch and Fluharty, 1998).

Overall, sort group had a modest impact on both finishing growth performance and carcass characteristics. Access to a high-energy diet for a longer period during the finishing phase, resulted in greater prevalence of liver abscesses and inedible livers; however, the effect of days on feed was confounded with BW at the start of the finishing phase. Limit-feeding a high-energy diet based on fermentable fiber in the growing phase had little carryover effect on finishing growth performance and carcass characteristics.

### Experiment 2

#### Growing phase growth performance and health

Composition of experimental diets was the same as in experiment 1, but 1.32LF diet was restricted to 2.2% of BW daily on a DM basis, and this amount was less than fed in experiment 1 where DM intake of the 1.32LF diet was about 2.4% of BW daily. In addition, gastrointestinal tract fill was equilibrated over a 14-d period at the end of the growing phase using a common diet (Table 1) for the equilibration.

Cattle performance during the growing phase is presented in Table 5. Average daily gains for 1.32LF2.2 were 15% lower (*P* < 0.01) than for 0.99AL, and the differences in treatment responses in experiments 1 and 2 can be attributed to the different amounts of 1.32LF that were offered. Gain:feed for heifers fed 1.32LF2.2 was 35% greater (*P* < 0.01) than for heifers receiving 0.99AL, and similarly NE_g_ concentration calculated from animal performance was greater (*P* < 0.01) for 1.32LF2.2 than for 0.99AL. The 0.99AL diet yielded 0.81 Mcal NE_g_/kg DM based on performance, whereas the 1.32LF2.2 diet yielded 1.27 Mcal NE_g_/kg DM. As for experiment 1, the observed:predicted ratio for dietary NE_g_ concentrations was greater for 1.32LF2.2 (0.96) than for 0.99AL (0.82). The larger deviation between observed and formulated NE for 0.99AL than for 1.32LF2.2 likely reflects the beneficial effects of intake restriction on nutrient digestion.

**Table 5.** Effect of ad libitum high-roughage or limit-fed high-energy diets in the backgrounding phase on performance and behavior (experiment 2)

Item	Diet^a^		SEM^b^	*P* value
	0.99AL	1.32LF2.2		
Number of pens	8	8		
Number of animals	186	184		
Body weight, kg				
Day 0	227.2	228.5	1.20	0.43
Day 14	246.5	239.3	1.17	<0.01
Day 28	267.8	255.9	1.22	<0.01
Day 42	287.4	273.6	1.47	<0.01
Day 56	306.7	288.1	1.60	<0.01
Day 70	330.7	309.8	1.31	<0.01
Day 84	342.9	325.5	2.62	<0.01
Treatment end^c^	343.7	327.3	2.68	<0.01
GIT equilibration, day 7^d^	340.8	335.6	1.70	0.05
GIT equilibration, day 14^d^	354.1	349.3	1.68	0.07
ADG, kg/d				
Days 0 to 14	1.38	0.77	0.09	<0.01
Days 0 to 28	1.45	0.98	0.05	<0.01
Days 0 to 42	1.43	1.07	0.04	<0.01
Days 0 to 56	1.42	1.07	0.04	<0.01
Days 0 to 70	1.48	1.16	0.02	<0.01
Days 0 to 84	1.38	1.16	0.03	<0.01
Days 0—treatment end^c^	1.33	1.13	0.03	<0.01
GIT equilibration, days 0 to 7^d^	-0.41	1.17	0.18	<0.01
GIT equilibration, days 7 to 14^d^	1.90	1.97	0.09	0.59
GIT equilibration, days 0 to 14^d^	0.75	1.57	0.10	<0.01
Day 0 to trial end^e^	1.25	1.19	0.02	0.06
Dry matter intake, kg/d				
Days 0 to 14	5.57	4.53	0.27	0.02
Days 0 to 28	6.90	5.05	0.32	<0.01
Days 0 to 42	7.93	5.33	0.36	<0.01
Days 0 to 56	8.77	5.54	0.38	<0.01
Days 0 to 70	9.37	5.72	0.38	<0.01
Days 0 to 84	9.68	5.96	0.33	<0.01
Day 0 to treatment end^c^	9.75	6.03	0.33	<0.01
GIT equilibration, days 0 to 7^d^	8.76	8.63	0.09	0.33
GIT equilibration, days 7 to 14^d^	8.68	8.58	0.05	0.18
GIT equilibration, days 0 to 14^d^	8.72	8.61	0.06	0.23
Day 0 to trial end^e^	9.60	6.37	0.29	<0.01
Daily intake, % of body weight daily				
Days 0 to 14	2.35	1.94	0.11	0.02
Days 0 to 28	2.79	2.08	0.13	<0.01
Days 0 to 42	3.08	2.12	0.13	<0.01
Days 0 to 56	3.28	2.15	0.14	<0.01
Days 0 to 70	3.36	2.13	0.14	<0.01
Days 0 to 84	3.40	2.15	0.11	<0.01
Day 0 to treatment end^c^	3.42	2.17	0.11	<0.01
GIT equilibration, days 0 to 7^d^	2.56	2.60	0.02	0.16
GIT equilibration, days 7 to 14^d^	2.50	2.51	0.01	0.57
GIT equilibration, days 0 to 14^d^	2.53	2.56	0.01	0.13
Day 0 to trial end^e^	3.30	2.20	0.10	<0.01
Gain:feed, kg/kg				
Days 0 to 14	0.260	0.170	0.02	<0.01
Days 0 to 28	0.219	0.194	0.01	0.11
Days 0 to 42	0.187	0.202	0.01	0.28
Days 0 to 56	0.166	0.192	0.01	0.06
Days 0 to 70	0.163	0.203	0.01	<0.01
Days 0 to 84	0.145	0.194	0.01	<0.01
Days 0 to treatment end^c^	0.139	0.188	0.01	<0.01
GIT equilibration, days 0 to 7^d^	-0.045	0.136	0.02	<0.01
GIT equilibration, days 7 to 14^d^	0.219	0.229	0.01	0.49
GIT equilibration, days 0 to 14^d^	0.087	0.183	0.01	<0.01
Day 0 to trial end^e^	0.132	0.187	0.004	<0.01
NE_m_, Mcal/kg dry matter^f^	1.39	1.91	0.04	<0.01
NE_g_, Mcal/kg dry matter^f^	0.81	1.27	0.03	<0.01
Morbidity, %				
Treated once or more	4.2	1.6	-	-
Treated twice or more	1.1	1.1	-	-
Treated 3 times (chronic)	1.1	1.1	-	-
Mortality, %^g^	0.5	0.5	-	-
Rumination, min/d^h^	455.7	302.8	12.01	<0.01
Activity, min/d^h^	346.2	369.5	3.12	<0.01

^a^0.99AL = 0.99 Mcal NE_g_/kg DM offered for ad libitum intake prior to gut-fill equilibration. 1.32LF2.2 = 1.32 Mcal NE_g_/kg DM limit-fed at 2.2% of BW on a DM basis.

^b^Largest SEM is reported.

^c^Treatment end date was day 84 for 2 blocks, and day 91 for 2 blocks.

^d^Gastrointestinal tract-fill equilibration diet was fed at 2.5% of BW daily on a DM basis for 14 d beginning on days 84 or 91 of the backgrounding phase. Day 0 of GIT equilibration was the same as treatment end on days 84 or 91.

^e^Trial end was after 98 days for 2 blocks and 105 days for 2 blocks.

^f^Net energy calculations from day 0 through gastrointestinal tract-fill equilibration phase using NRC (1996) equations.

^g^Does not include chronics (heifers removed from study after 3 treatments).

^h^Measured using 3-axial accelerometer ear tags (Allflex Livestock Intelligence, Madison, WI).

Cattle fed 0.99AL consumed more (*P* < 0.01) DM than 1.32LF2.2 cattle, except by design during gastrointestinal tract-fill equilibration where DM intake did not differ (*P* = 0.23). Cattle that had been fed 0.99AL lost BW during the first 7 d of the equilibration period, which likely reflects a decrease in gut fill in response to the reductions in feed intake and dietary forage concentration. During the 14-d equilibration phase, cattle that had been fed 1.32LF2.2 gained weight faster than during the treatment application, which likely reflects an increase in gut fill in response to increased DM intake and a higher roughage concentration in the equilibration diet than in the 1.32LF diet. The different responses of cattle fed 0.99AL compared to those fed 1.32LF2.2 signify the importance of this period for accuracy and comparability of performance parameters across dietary treatments, particularly when roughage-based diets are fed for ad libitum intake (Watson et al., 2013).

Cattle were generally healthy and required little antimicrobial intervention (Table 5). Mortality was also low with a single death for each treatment (Table 5). A generally accepted and beneficial practice for newly received, commingled cattle is to mass-medicate an entire group of cattle with an antibiotic to reduce incidence of disease (Lofgreen et al., 1980; Munoz et al., 2020; Word et al., 2020). Metaphylactic use of tulathromycin on arrival may have minimized the disease load, contributing to the limited numbers of morbid and dead heifers.

#### Rumination and activity

**Figure 3.**
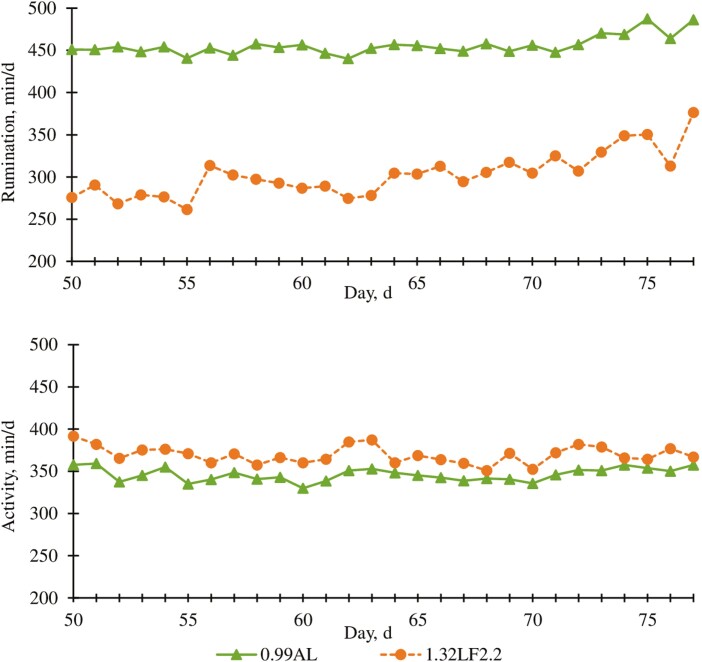
Effect of ad libitum high-roughage or limit-fed high-energy diets in the backgrounding phase on daily rumination and activity (experiment 2). 0.99AL (filled triangle) = 0.99 Mcal NE_g_/kg DM offered for ad libitum intake, *n* = 186; 1.32LF2.2 (filled circle) = 1.32 Mcal NE_g_/kg DM limit-fed at 2.2% of BW daily, *n* = 184. Rumination (top graph): diet, *P* < 0.0001; day, *P* < 0.0001; diet × day, *P* = 0.04. SEM = 15.9. Activity (bottom graph): diet, *P* < 0.001; day, *P* = 0.01; diet × day, *P* = 0.93. SEM = 9.5.

**Figure 4.**
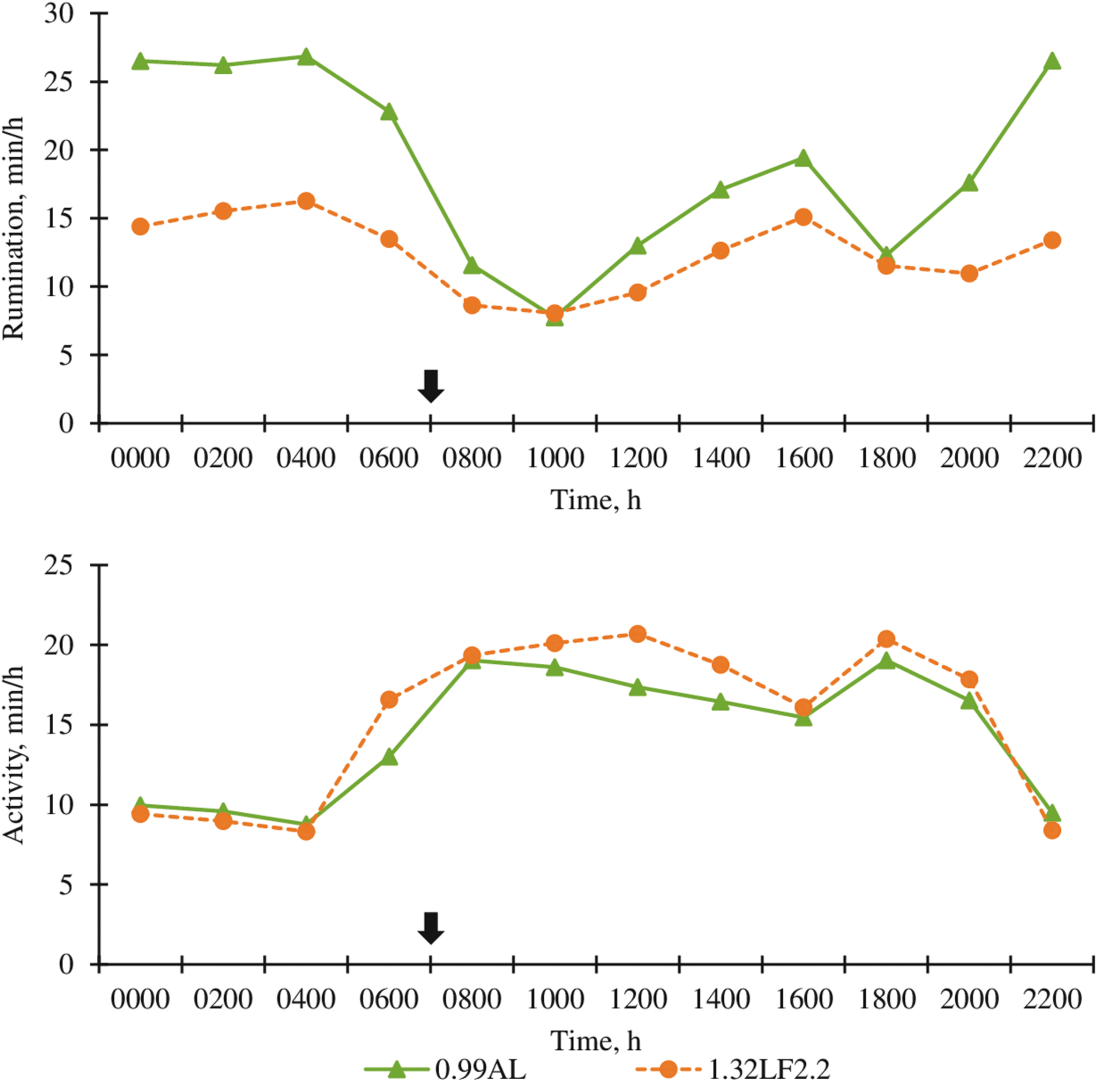
Effect of ad libitum high-roughage or limit-fed high-energy diets in the backgrounding phase on hourly rumination and activity (experiment 2). 0.99AL (filled triangle) = 0.99 Mcal NE_g_/kg DM offered for ad libitum DM intake, *n* = 186; 1.32LF2.2 (filled circle) = 1.32 Mcal NE_g_/kg DM limit-fed at 2.2% of body weight daily, *n* = 184. Arrows represent time of feeding (0700 hours). Rumination (top graph): diet, *P *< 0.0001; hour, *P* < 0.0001; diet × hour, *P* < 0.0001. SEM = 0.59. Activity (bottom graph): diet, *P* < 0.0001; hour, *P* < 0.0001; diet × hour, *P* < 0.0001. SEM = 0.33.

Cattle fed 1.32LF2.2 spent 154 min/d less ruminating than 0.99AL contemporaries (*P* < 0.01; Table 5, Fig. 3). Similar to experiment 1, differences in time spent ruminating were likely associated with roughage concentrations and particle size of each diet. A dietary treatment × day interaction (*P* = 0.04; Fig. 3) was detected for rumination; time spent ruminating increased slightly between days 50 and 77 for cattle fed 1.32LF, whereas cattle fed 0.99AL appeared to have rumination times that were stable between days 50 and 77. A dietary treatment × hour interaction (*P* < 0.01; Fig. 4) was detected for rumination; 0.99AL cattle spent more time ruminating overnight than 1.32LF2.2 cattle (2000 to 0600 hours; *P* < 0.05), but no differences (*P* > 0.10) were observed between treatments at 1000 hours when rumination time for both groups reached a nadir. Cattle fed 1.32LF2.2 demonstrated more (*P* < 0.01; Fig. 3) activity than cattle fed 0.99AL, but the differences were relatively small. A dietary treatment × hour interaction (*P* < 0.01; Fig. 4) was detected for activity, but this effect appears to be nuanced, with 1.32LF2.2 cattle being slightly more active than 0.99AL cattle 1 h before feeding and again 3 h to 7 h after feeding, but without differences between the treatments at other times of the day.

Unlike experiment 1 where no differences in activity were observed between treatments, 1.32LF2.2 cattle were more active than 0.99AL cattle in experiment 2. Perhaps this was due to greater hunger of cattle fed 1.32LF in experiment 2 due to treatment design differences; limit-fed cattle were offered more feed in experiment 1 than in experiment 2. However, it should also be pointed out that the statistically significant effects of treatment on activity in experiment 2 were nonetheless small. One other factor that differed between experiments 1 and 2 was pen size, which was larger in experiment 2 than experiment 1; however, this might be expected to affect both treatments similarly, because all pens within a single trial were of identical size. Our results demonstrated that cattle limit-fed a high-energy diet required less time to ruminate but were slightly more active than cattle fed a forage-based diet for ad libitum intake.

#### Finishing phase growth performance and carcass characteristics

Finishing growth performance is presented in Table 6. A significant interaction (*P* = 0.03) between growing diet and sort group was observed for mortality, because 1.32LF2.2 cattle had greater mortality in the light-sort group than the heavy-sort group, whereas 0.99AL cattle did not demonstrate differences between sort groups for mortality. No other significant interactions between growing diet or sort group were observed in this experiment. Morbidity was 15.5% greater for 1.32LF2.2 than for 0.99AL. Initial BW tended (*P* = 0.06) to be greater for 1.32LF2.2 than for 0.99AL. No differences (*P* ≥ 0.53) between growing diets were observed for days on feed, final BW, or ADG. Light-sort cattle had greater (*P* = 0.01) morbidity than heavy-sort cattle. Heavy-sort cattle had greater initial BW (*P *< 0.01), fewer days on feed (*P* < 0.01), and better ADG (*P* < 0.01) than light-sort cattle. Sort group did not affect (*P* = 0.51) final BW.

**Table 6.** Effect of dietary treatment during the backgrounding phase and sort group during the finishing phase on finishing growth performance and carcass characteristics (experiment 2)

	Sort Group^1^							
	Heavy		Light					
	Backgrounding Diet^2^					*P* value^4^		
Item	0.99AL	1.32LF2.2	0.99AL	1.32LF2.2	SEM^3^	S	B	S × B
Number of pens	1	1	1	1				
Finishing phase performance								
Number of animals	94	91	92	92				
Days on feed, d	144	144	200	200	0.5	<0.01	0.99	0.99
Initial weight, kg	386.9	381.7	336.8	332.8	2.6	<0.01	0.06	0.78
Final weight^5^, kg	603.1	601.7	602.5	595.4	6.4	0.51	0.53	0.59
ADG, kg/d	1.51	1.53	1.33	1.31	0.03	<0.01	0.90	0.43
Dry matter intake, kg/d	9.97	9.44	8.66	8.69	—	—	—	—
Gain:feed, kg/kg	0.151	0.162	0.154	0.151	—	—	—	—
Morbidity, %	5.3	16.0	10.4	30.6	4.5	0.01	<0.01	0.19
Mortality, %	2^ab^	0^a^	1^a^	5^b^	1.3	0.14	0.46	0.03
Carcass traits								
Number of animals	92	88	88	83				
Hot carcass weight, kg	385.7	385.5	384.3	385.5	4.0	0.83	0.91	0.84
Dressing percentage, %	63.95	64.07	63.78	64.74	—	—	—	—
Backfat, cm	1.77	1.79	1.91	1.89	0.06	0.02	0.97	0.74
USDA yield grade	2.58	2.65	2.83	2.85	0.10	0.01	0.62	0.80
Marbling score^6^	540	531	523	528	17.4	0.39	0.84	0.56
Ribeye area, cm^2^	96.6	94.9	93.8	94.1	1.1	0.09	0.52	0.32
USDA quality grade, %								
Select	4.8	6.4	8.8	5.1	3.1	0.57	0.65	0.26
Choice	86.4	83.7	81.9	87.5	3.5	0.92	0.67	0.24
Prime	8.9	8.8	9.4	6.5	3.4	0.74	0.59	0.62

^a, b^Least square means in the same row with different superscripts are significantly different (*P* < 0.05).

^1^Sort groups for each backgrounding treatment were created prior to finishing phase. Heavy-sort groups were harvested after 144 d of finishing, and light-sort groups were harvested after 200 d of finishing.

^2^Diets offered during the backgrounding phase prior to the finishing phase. 0.99AL = 0.99 Mcal NE_g_/kg DM offered for ad libitum intake. 1.32LF2.2 = 1.32 Mcal NE_g_/kg DM limit-fed at 2.2% of BW daily on a DM basis. Both dietary treatment groups had a 14-d gut-fill equilibration period before initiation of the finishing phase.

^3^Largest standard error of the means are reported.

^4^S = sort group; B = backgrounding diet; S × B = sort group × backgrounding diet interaction.

^5^Final weight was calculated from hot carcass weight divided by average dressing percentage of each finishing pen.

^6^<400 = Select. 400 to 499 = low Choice. 500 to 599 = average Choice. 600 to 699 = high Choice.

Carcass characteristics for experiment 2 are presented in Table 6. Hot carcass weights were not affected by growing diet or sort group. No effects (*P* > 0.52) of growing diet were observed for any carcass characteristics. Carcasses from heavy-sort heifers had less (*P* = 0.02) backfat and smaller (*P* = 0.01) USDA yield grade scores compared with carcasses from light-sort heifers. Carcasses from heavy-sort heifers also tended (*P* = 0.09) to have greater ribeye areas compared with carcasses from light-sort heifers. Marbling score and USDA quality grades were not affected (*P* ≥ 0.39) by backgrounding diet.

Similar to experiment 1, morbidity in the finishing phase was affected by sort group, with greater sickness in light-sort cattle compared to heavy-sort cattle. In contrast to experiment 1, there was also an effect of growing diet on morbidity. One possible reason for this effect could be that intake restriction of the limit-fed, high-energy diet was numerically greater in the growing phase of experiment 2 than experiment 1. This may have contributed to a greater change in intakes when cattle previously limit-fed were transitioned to an ad libitum diet at the feedlot.

A recent study modeled potential economic benefits of what is termed “progressive limit feeding,” whereby newly received feedlot cattle receive a short period of restricted intake to maintain constant metabolic body size, followed by ad libitum feeding for the rest of the finishing period (Hannon and Murphy, 2019). Limit-fed animals experience compensatory gains due to gain efficiency and reduced metabolic activity of various organs, but it is not known how long efficiency effects from limit-feeding last into the ad libitum feeding period (Hannon and Murphy, 2019). Several studies suggest carryover effects can improve gain:feed if cattle are limit-fed during the growing phase and subsequently placed on an ad libitum nutritional plane until final market weight is reached (Drouillard et al., 1991; Sainz et al., 1995; Knoblich et al., 1997; Reinhardt et al., 1998). In experiment 1, cattle fed the 1.32LF85% diet during the growing phase had lower ADG than 0.99AL cattle in the finishing phase, whereas in experiment 2 finishing phase ADG was not affected by growing phase diet. In both experiments 1 and 2, gain:feed during the finishing phase was generally similar between growing diets.

In contrast to Sainz et al. (1995), in experiment 2 our cattle were fed a gastrointestinal tract-fill equilibration diet for 2 wk prior to arrival at the feedlot for finishing, which may have reduced any compensatory gain that 1.32LF2.2 cattle otherwise would have realized. In addition, the supply of energy in our experiment was similar between 1.32LF2.2 and 0.99AL, as demonstrated by BW at the end of the growing phase being similar between treatments after the 14-d equilibration period. A meta-analysis of growing diets and subsequent finishing diets revealed that ad libitum feeding in the finishing phase effectively diminishes differences in final carcass quality regardless of previous plane of nutrition, including cattle limit-fed during the backgrounding growth phase (Lancaster et al., 2014). Our carcass results from experiment 2 support this conclusion. Sip and Pritchard (1991) observed no differences in carcasses of steers limit-fed growing diets formulated to provide NE intakes similar to diets fed for ad libitum intake, then transitioned to an ad libitum finishing diet for 94 d.

Although sort group in the finishing phase can affect finishing growth performance and carcass characteristics to some degree, growing-phase treatment had little carryover effect on finishing performance and carcass characteristics after a long finishing period in which cattle were offered high-energy diets ad libitum. This agrees with observations from experiment 1.

### Experiment 3—Intake and Digestibility Study

By design, intakes of DM, organic matter, neutral detergent fiber, and acid detergent fiber were less (*P* < 0.01; Table 7) for 1.32LF85% than for 0.99AL. Conversely, also by design, intake of starch was greater (*P* < 0.01) for heifers fed 1.32LF85% than for cohorts fed 0.99AL. Cattle in this experiment served as their own reference for determining ad libitum intake, which differs slightly from experiment 1 where each pen of heifers fed 1.32LF85% was paired with a pen of heifers fed 0.99AL to determine the amount fed. Although DM intake of the high-energy diet was restricted, energy intake (based on formulated NE_g_ concentrations) was greater for 1.32LF85% cattle than for those receiving 0.99AL, suggesting that feed offered to the 1.32LF85% cattle may have been near ad libitum intake.

**Table 7.** Effect of feeding a high-roughage diet for ad libitum intake or a high-energy diet at 85% of ad libitum intake on diet digestibility and ruminal characteristics (experiment 3)

	Diet^a^			
Item	0.99AL	1.32LF85%	SEM^b^	*P* value
Number of observations	8	8		
Intake, kg/d				
Dry matter	8.06	6.23	0.37	<0.01
Organic matter	7.36	5.88	0.35	<0.01
Neutral detergent fiber	2.92	1.54	0.11	<0.01
Acid detergent fiber	1.47	0.62	0.05	<0.01
Starch	0.95	2.05	0.08	<0.01
Apparent total-tract digestibility, %				
Dry matter	74.8	78.7	0.77	0.01
Organic matter	77.1	82.0	0.62	<0.01
Neutral detergent fiber	73.4	73.5	1.45	0.94
Acid detergent fiber	67.6	66.4	1.54	0.59
Starch	94.4	96.2	1.16	0.32
TDN,^c^ %	72.5	80.2	0.58	<0.01
DE,^c^ Mcal/kg dry matter	3.19	3.54	0.026	<0.01
ME,^c^ Mcal/kg dry matter	2.68	2.97	0.022	<0.01
NE_m_,^c^ Mcal/kg dry matter	1.76	2.00	0.018	<0.01
NE_g_,^c^ Mcal/kg dry matter	1.14	1.35	0.016	<0.01
Ruminal ammonia,^d^ m*M*	5.22	3.89	0.49	0.03
Ruminal volatile fatty acids^d^				
Total, m*M*	109.37	82.81	5.02	<0.01
Acetate, m*M*	66.90	44.18	2.48	<0.01
Propionate, m*M*	23.77	24.63	2.20	0.63
Butyrate, m*M*	13.78	9.05	0.53	<0.01
Valerate, m*M*	2.24	2.62	0.38	0.42
Isobutyrate, m*M*	0.89	0.67	0.04	<0.01
Isovalerate, m*M*	1.66	1.65	0.24	0.98
Acetate:propionate	2.80	1.98	0.15	<0.01
Liquid passage rate, %/h	11.3	5.7	1.04	<0.01
Ruminal liquid volume, L	32.6	48.2	3.86	<0.01

^a^0.99AL = 0.99 Mcal NE_g_/kg DM fed for ad libitum intake. 1.32LF85% = 1.32 Mcal NE_g_/kg DM limit-fed at 85% of 0.99AL treatment DM intake.

^b^Largest SEM is reported.

^c^TDN = total digestible nutrients; DE = digestible energy; ME = metabolizable energy. Calculated from diet digestibilities according to NASEM (2016) assuming fat digestibility was 60%.

^d^Average of values collected at 0, 2, 4, 6, 8, 12, 18, and 24 h after feeding.

Apparent total-tract diet digestibilities of DM and organic matter were 5.2% and 6.4% greater (*P* < 0.01; Table 7), respectively, for cattle fed 1.32LF85% than for those 0.99AL. At the same time, digestibilities of neutral detergent fiber (*P* = 0.94), acid detergent fiber (*P* = 0.59), and starch (*P* = 0.32) were unaffected by treatment. Better digestibilities of DM and organic matter for 1.32LF85% than 0.99AL agrees with observations of Spore et al. (2019), and this reflects changes in dietary ingredients (1.32LF85% contained more corn and less forage than 0.99AL) as well as lower intakes. The lack of difference between treatments for fiber digestibility may reflect several factors working in opposite directions. The lower feed intake for 1.32LF85% than for 0.99AL might be expected to improve fiber digestion as a consequence of slower ruminal passage rates. The fiber composition of the diets also differed; both diets contained 40% wet corn gluten feed on a DM basis, but fiber in wet corn gluten feed represented a greater portion of total fiber in the 1.32LF85% diet compared to the 0.99AL diet. Because fiber in wet corn gluten feed is more digestible than fiber provided by roughages (NASEM, 2016), the fiber composition would be expected to favor fiber digestion for 1.32LF85% over 0.99AL. However, fiber-digesting ruminal bacteria may have been more active for cattle fed 0.99AL than for those fed 1.32LF85%, offsetting the other factors. Similar to fiber, starch digestion was unaffected by diet; this can be attributed to the similar source of starch in both diets (corn).

Cattle fed 1.32LF85% had slower ruminal liquid dilution rates (*P* < 0.01; Table 7) than those fed 0.99AL, but ruminal liquid volume was greater (*P* < 0.01) for 1.32LF85% than for 0.99AL. Limit-feeding a high-energy diet could impact site of digestion by changing the rate at which nutrients flow through the gastrointestinal tract. Spore et al. (2019) also demonstrated limit-fed, high-energy diets fed for programmed rates of gain led to a slower liquid passage rate than lower-energy diets fed for ad libitum intake. Other reports demonstrate decreases in liquid passage rate when cattle are given diets with greater energy concentrations at restricted intakes (Galyean et al., 1979; Murphy et al., 1994b). In opposition to these results, Clark et al. (2007) observed no statistically significant differences in liquid passage rate when diet intakes were restricted to 80% of ad libitum intakes, although liquid dilution rate was numerically less for cattle restricted to 80% of intake (6.8%/h) than for those fed for ad libitum intake (7.7%/h).

**Figure 5.**
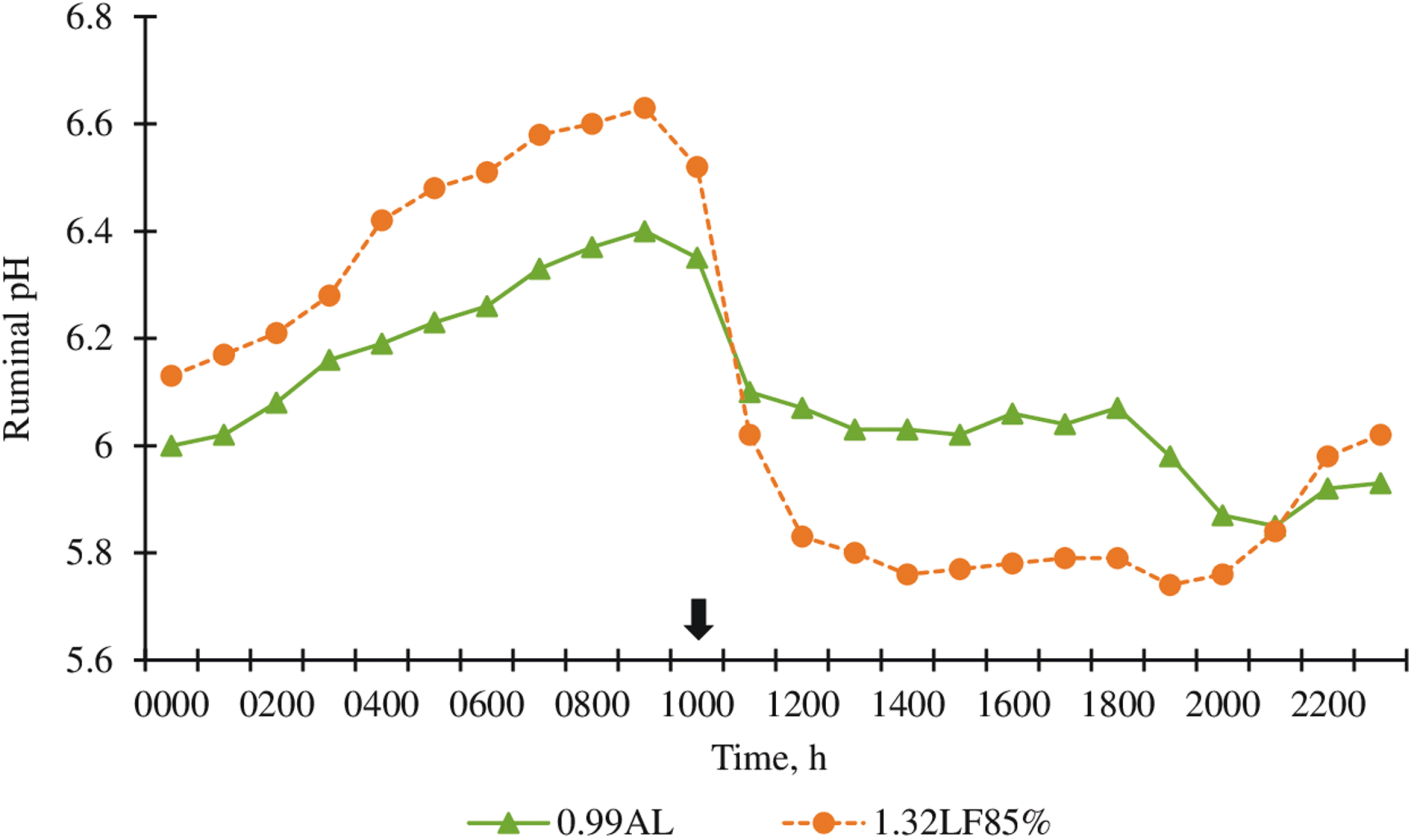
Effect of ad libitum high-roughage or limit-fed high-energy diets on hourly pH (experiment 3). 0.99AL (filled triangle) = 0.99 Mcal NE_g_/kg DM offered for ad libitum intake, *n* = 7; 1.32LF85% (filled circle) = 1.32 Mcal NE_g_/kg DM limit-fed at 85% of 0.99AL DM intake, *n* = 8. The arrow represents time of feeding (1000 hours). Diet, *P* = 0.93; hour, *P* < 0.0001; diet × hour, *P* < 0.0001. SEM = 0.11.

Greater ruminal liquid volumes in cattle fed 1.32LF85% than in cattle fed 0.99AL were unexpected and contradicts the findings of Murphy et al. (1994a) who reported limit-fed cattle had lower ruminal fill volumes than full-fed cohorts. Two potential explanations for this response include differences in meal-intake patterns and ruminal osmotic pressures; lower pH associated with rapidly fermented carbohydrates and organic acid production can increase ruminal osmotic pressure, elevating liquid fill volume (Howard, 1981). Moreover, our experiment was conducted during the heat of summer in mid-July, which might alter water intakes. In contrast to our results, Montgomery et al. (2004) found cattle limit-fed diets based on wet corn gluten feed had greater ruminal liquid fill 4 to 8 h after feeding, compared to diets based on steam-flaked corn. It should be noted that the methodology we used to measure liquid passage rates could be differentially affected by the treatments; the methodology is designed for use with steady state conditions, which were better demonstrated for 0.99AL than for 1.32LF85% (e.g., Fig. 5).

A dietary treatment × hour interaction (*P* < 0.01; Fig. 5) was detected for ruminal pH. Ruminal pH declined more rapidly between feeding and 2 h after feeding for 1.32LF85% than for 0.99AL, and it remained lower for 1.32LF85% than for 0.99AL through 10 h after feeding. Subsequently, between 8 and 10 h after feeding, ruminal pH increased more for 1.32LF85% than for 0.99AL and remained greater for 1.32LF85% than for 0.99AL until feeding. These patterns for ruminal pH can be readily explained by the different diet compositions and within-day feed intake patterns. At time points soon after feeding, cattle fed 1.32LF85% consumed more DM from a more digestible diet compared to 0.99AL, which would lead to the more rapid decline in pH post-feeding. At time near 8 h after feeding, when cattle fed 1.32LF85% had no feed remaining, cattle fed 0.99AL consumed additional meals that led to the lower ruminal pH at times more than 12 h after feeding. Our observations generally follow those of Spore et al. (2019) with similar types of feed restriction. Intake of all-concentrate or starch-rich diets, especially processed grains, leads to decreases in ruminal pH (Murphy et al., 1994a). Murphy et al. (1994a) observed steers with ad libitum intake of all-concentrate diets had lower ruminal pH during the first 2 h after feeding compared to cattle limit-fed at 70% of ad lib intakes. In the present experiment, greater dietary energy concentration in the 1.32LF85% diet than the 0.99AL diet would have contributed to the greater decline in ruminal pH, although intake patterns were likely to also have played a role.

**Figure 6.**
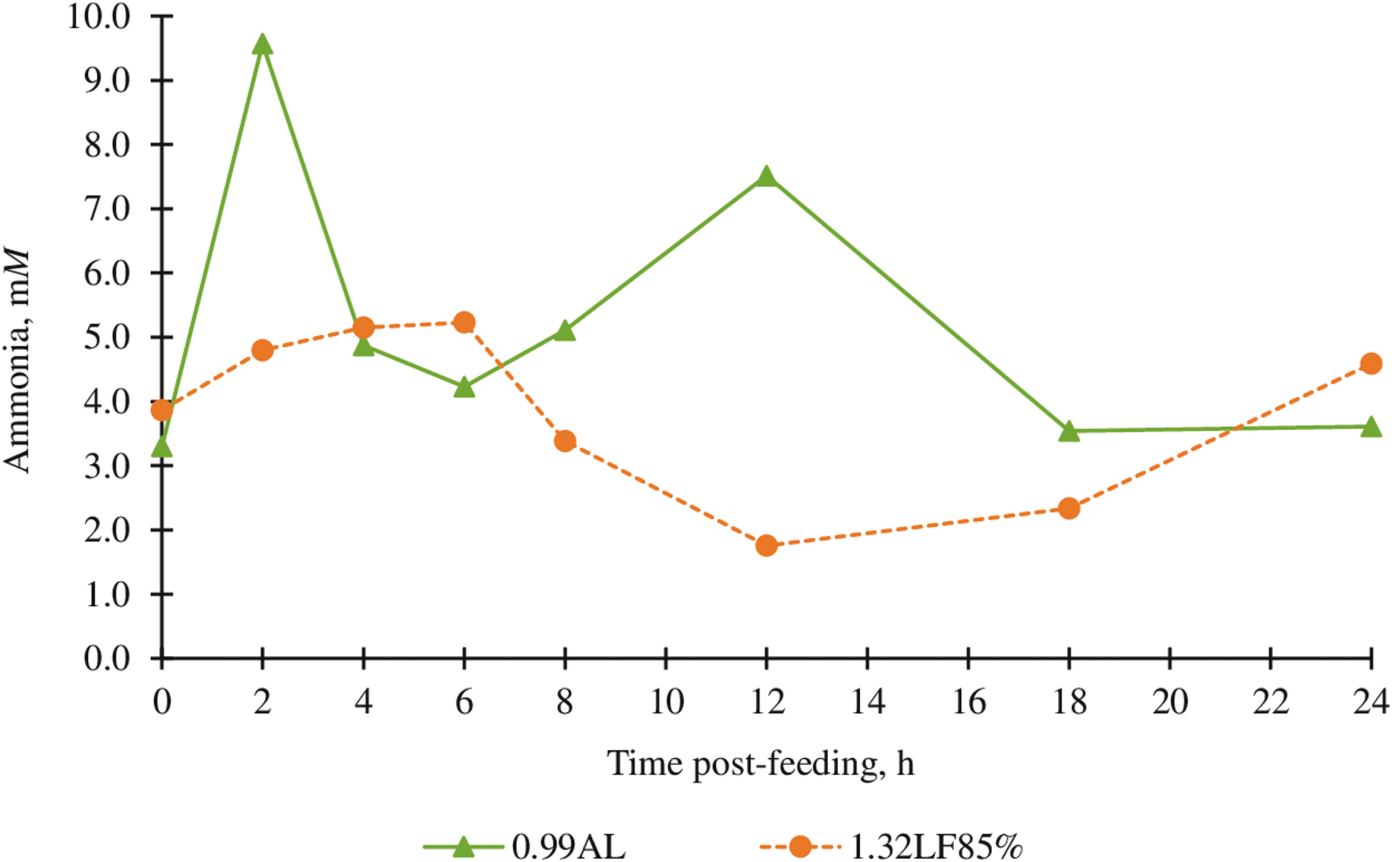
Effect of ad libitum high-roughage or limit-fed high-energy diets on ruminal ammonia concentrations (experiment 3). 0.99AL (filled triangle) = 0.99 Mcal NE_g_/kg DM offered for ad libitum intake, *n* = 7; 1.32LF85% (filled circle) = 1.32 Mcal NE_g_/kg DM offered at 85% of 0.99AL DM intake, *n* = 8. diet, *P* = 0.03; hour, *P* < 0.0001; diet × hour, *P* < 0.0001. SEM = 0.73.

Ruminal ammonia concentrations were greater (*P* = 0.03; Table 7) for 0.99AL compared with 1.32LF85%, but there was also a dietary treatment × hour interaction (*P* < 0.01; Fig. 6), with cattle fed 0.99AL having greater concentrations of ammonia 2 h after feeding and again from 8 to 18 h after feeding compared with cattle fed 1.32LF85%. Ruminal ammonia concentrations over time likely reflect meal-eating behavior differences between the diets; cattle fed 1.32LF85% consumed daily rations within a few hours after feeding, whereas cattle fed 0.99AL returned for additional meals later in the day. Differences in overall ruminal ammonia concentrations were likely associated with diet composition. Crude protein concentrations were 2.5% greater in 0.99AL compared with 1.32LF85%. In addition, 0.99AL likely contained more ruminally degradable protein compared with 1.32LF85%. These data contradict Murphy et al. (1994b) who fed corn-silage-based diets for ad libitum, 90%, or 80% of ad libitum intakes. They found that restricted intakes led to greater ruminal ammonia concentration, perhaps due to less microbial protein synthesis or less dilution of ammonia from ruminal liquid fill. However, unlike our experiment, Murphy et al. (1994b) did not modify dietary composition as intake was restricted. In our experiment, ruminal liquid fill was greater for 1.32LF85% than 0.99AL, which may have also contributed to differences in ammonia concentrations. Ruminal ammonia concentrations were also greater for steers limit-fed wet corn gluten feed (Montgomery et al., 2004; Spore et al., 2019) compared to those fed diets with greater roughage inclusion. In our study, wet corn gluten feed was 40% of diet DM in both diets, but it would have provided a slightly larger percentage of the dietary protein for 1.32LF85% than for 0.99AL. For our experimental diets, ammonia concentrations appeared sufficient to optimize ruminal fermentation and support microbial growth (Satter and Slyter, 1974), reflecting adequate degradable protein in the diets.

**Figure 7.**
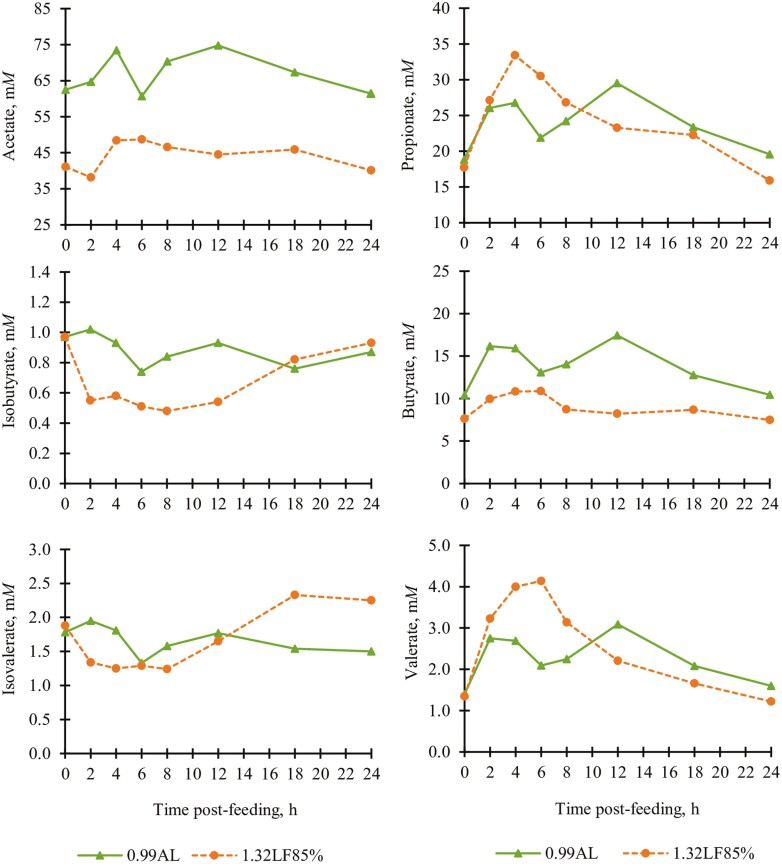
Effect of ad libitum high-roughage or limit-fed high-energy diets on ruminal VFA concentrations (experiment 3). 0.99AL (filled triangle) = 0.99 Mcal NE_g_/kg DM offered for ad libitum intake, *n* = 7; 1.32LF85% (filled circle) = 1.32 Mcal NE_g_/kg DM offered at 85% of 0.99AL DM intake, *n* = 8. Acetate: diet, *P* < 0.0001; hour, *P* < 0.01; diet × hour, *P* = 0.21. Propionate: diet, *P* = 0.63; hour, *P* < 0.0001; diet × hour, *P* < 0.001. Isobutyrate: diet, *P* < 0.0001; hour, *P* < 0.0001; diet × hour, *P* < 0.0001. Butyrate: diet, *P* < 0.0001; hour, *P* < 0.0001; diet × hour, *P* < 0.01. Isovalerate: diet, *P* = 0.98; hour, *P* = 0.04; diet × hour, *P* < 0.01. Valerate: diet, *P* = 0.42; hour, *P* < 0.0001, diet × hour, *P* < 0.0001.

Cattle fed 0.99AL had greater (*P* < 0.01; Table 7) concentrations of total ruminal VFA than cattle receiving 1.32LF85%, and this was largely a result of greater (*P* < 0.01) concentrations of acetate. Butyrate was also greater (*P* < 0.01) for 0.99AL compared with 1.32LF. Propionate, isovalerate, and valerate did not differ (*P* > 0.10) between treatments. There were dietary treatment × hour interactions (*P* < 0.01; Fig. 7) for propionate, butyrate, and valerate, where all three interactions reflected meal-eating behavior similarly demonstrated by ammonia concentrations; concentrations peaked twice for cattle fed 0.99AL at 2 h and again 12 h after feeding, whereas cattle fed 1.32LF85% had only a single peak at 4 to 6 h after feeding. There were also dietary treatment × hour interactions (*P* < 0.01; Fig. 7) for isobutyrate and isovalerate, with greater decreases in concentration 2 h after feeding for 1.32LF85% than 0.99AL. Acetate:propionate ratio was lower (*P* < 0.01) for 1.32LF85% compared with 0.99AL. Molar proportions of acetate were greater (*P* < 0.01) for 0.99AL cattle, whereas proportions of propionate, isobutyrate, isovalerate, and valerate were greater (*P* < 0.01) for 1.32LF85% cattle (data not shown). Total ruminal VFA concentrations of 1.32LF85% cattle were expected to be greater than those of 0.99AL cattle soon after feeding, because intake of fermentable substrate was presumably greater within the first 4 h after feeding. However, even when the cattle fed 1.32LF85% reached peak VFA concentrations at 4 h after feeding (Fig. 7), total VFA concentrations were greater for 0.99AL than for 1.32LF85%; this may reflect that greater ruminal liquid volume in 1.32LF cattle than 0.99AL cattle could have diluted VFA or perhaps a faster rate of VFA absorption for cattle fed 1.32LF85% in response to the lower pH during this time frame (Fig. 5). Lower total VFA concentration in 1.32LF85% cattle than 0.99AL cattle observed in this experiment agrees with Spore et al. (2019). Clark et al. (2007) also observed lower concentrations of total ruminal VFA when feeding a high-energy diet at 80% of ad libitum intake compared to ad libitum intake. A lower acetate:propionate ratio in 1.32LF85% cattle than 0.99 AL cattle can be attributed to differences in diet composition, with greater intake of starch for cattle fed high-energy, limit-fed diets than cattle fed roughage-based diets fed for ad libitum intakes. As a result of a lower acetate:propionate ratio for 1.32LF85% than for 0.99AL, less hydrogen would presumably be available for methane production (Russell, 1998) and, thus, less energy should have been lost as methane by cattle fed 1.32LF85% than those fed 0.99AL; this helps explain in part the differences observed for dietary NE concentrations in experiments 1 and 2.

Dietary NE_g_ and NE_m_ concentrations, calculated from digestion (Table 7), were greater (*P* < 0.01) for 1.32LF85% than 0.99AL, similar to observations from experiments 1 and 2 based on growth performance (Tables 3 and 5). Estimated dietary NE_g_ of 0.99AL, calculated from digestion, was 1.14 Mcal NE_g_/kg DM (Table 7), a value 15% greater than estimated from tabular values of feed ingredients (NASEM, 2016); this contrasts with NE_g_ concentrations based on performance for both experiments 1 and 2 (0.68 and 0.81 Mcal NE_g_/kg DM, respectively) that were lower than NASEM (2016) predictions. Relative to estimates from book values (NASEM, 2016), the NE_g_ of the 1.32LF85% diet was quite close to the formulation when calculated from digestibilities (1.35 Mcal NE_g_/kg DM), whereas NE_g_ based on performance was a little lower than formulation in experiment 2 (1.27 Mcal NE_g_/kg DM) and much lower in experiment 1 (1.01 Mcal NE_g_/kg DM). The differences in dietary NE_g_ between formulations based on book values and values estimated from animal performance can be affected by a myriad of factors such as environment, cattle phenotype, and quality of feed ingredients. For 0.99AL, the estimate of NE_g_ based on performance was less than that based on digestion, and this suggests that the efficiency of digestible energy utilization for that diet was overestimated by NASEM (2016) equations.

## Conclusions

Limit-feeding high-energy diets during the growing phase led to 47% (experiment 1) and 35% (experiment 2) better gain:feed and 5.2% greater DM diet digestibility (experiment 3) compared to traditional ad libitum forage-based growing strategies. Health during the growing period was not affected by dietary treatments. Heifers fed 1.32LF85% had greater marbling scores and thicker backfat, as measured by ultrasound, at the end of the growing phase compared with heifers fed 0.99AL; differences in backfat remained following the finishing phase, but there was minimal effect of previous growing diet strategy on marbling scores at slaughter. Limit-feeding high-energy diets based on corn and fermentable fiber during the growing phase did not increase prevalence of liver abscesses. In some instances, sort-group did affect performance and carcass characteristics. Liver condemnation was greater in light-sort heifers compared with heavy-sort heifers which may have resulted from increased days on feed or the trend for greater morbidity for light heifers compared with heavy heifers. Greater gain efficiency and diet digestibility in cattle limit-fed high-energy diets used in our experiment can provide beef producers with a strategy to boost operational productivity and reduce manure output.

In these experiments, we evaluated two separate approaches to limit-feeding growing heifers. In experiment 1, limit-fed heifers were fed at 85% of ad libitum intake heifers. This approach is likely not practical on a commercial scale as it would require a group of cattle fed for ad libitum intake to determine limit-fed feed deliveries. In experiment 2, limit-fed heifers were fed at 2.2% of BW daily (DM basis). To accomplish this, weekly pen weights were measured which also would not be practical on a commercial scale. A more reasonable approach to limit-feeding growing cattle may be to determine BWs at the start of the feeding period, feed at a designated % of BW, and use predicted weight gains to increase feed delivery over time.
